# Genomes in turmoil: quantification of genome dynamics in prokaryote supergenomes

**DOI:** 10.1186/s12915-014-0066-4

**Published:** 2014-08-21

**Authors:** Pere Puigbò, Alexander E Lobkovsky, David M Kristensen, Yuri I Wolf, Eugene V Koonin

**Affiliations:** National Center for Biotechnology Information, National Library of Medicine, National Institutes of Health, Bethesda, MD 20894 USA

## Abstract

**Background:**

Genomes of bacteria and archaea (collectively, prokaryotes) appear to exist in incessant flux, expanding via horizontal gene transfer and gene duplication, and contracting via gene loss. However, the actual rates of genome dynamics and relative contributions of different types of event across the diversity of prokaryotes are largely unknown, as are the sizes of microbial supergenomes, i.e. pools of genes that are accessible to the given microbial species.

**Results:**

We performed a comprehensive analysis of the genome dynamics in 35 groups (34 bacterial and one archaeal) of closely related microbial genomes using a phylogenetic birth-and-death maximum likelihood model to quantify the rates of gene family gain and loss, as well as expansion and reduction. The results show that loss of gene families dominates the evolution of prokaryotes, occurring at approximately three times the rate of gain. The rates of gene family expansion and reduction are typically seven and twenty times less than the gain and loss rates, respectively. Thus, the prevailing mode of evolution in bacteria and archaea is genome contraction, which is partially compensated by the gain of new gene families via horizontal gene transfer. However, the rates of gene family gain, loss, expansion and reduction vary within wide ranges, with the most stable genomes showing rates about 25 times lower than the most dynamic genomes. For many groups, the supergenome estimated from the fraction of repetitive gene family gains includes about tenfold more gene families than the typical genome in the group although some groups appear to have vast, ‘open’ supergenomes.

**Conclusions:**

Reconstruction of evolution for groups of closely related bacteria and archaea reveals an extremely rapid and highly variable flux of genes in evolving microbial genomes, demonstrates that extensive gene loss and horizontal gene transfer leading to innovation are the two dominant evolutionary processes, and yields robust estimates of the supergenome size.

**Electronic supplementary material:**

The online version of this article (doi:10.1186/s12915-014-0066-4) contains supplementary material, which is available to authorized users.

## Background

Comparative genomics of bacteria and archaea (collectively, prokaryotes) reveals extensive variation of gene repertoires, which is thought to reflect a highly dynamic regime of genome evolution [1–6]. Prokaryotic genomes present a wide variety of genome sizes, from approximately 150 kb in some intracellular endosymbionts of insects [7] to approximately 13 Mb in the largest cyanobacteria [8] and myxobacteria [9]. This broad range of genome sizes is reflected in the diversity of gene repertoires: only a tiny minority of prokaryotic genes are (nearly) universal whereas the great majority are present in small subsets of genomes [6,10,11]. Substantial differences in genome size and gene content are often evident between species within the same genus [12–14] and even between strains of the same species [15–17].

The enormous diversity of the gene repertoires among bacteria and archaea implies that prokaryotic genomes exist in a state of incessant flux, expanding through horizontal gene transfer (HGT), gene duplication and possibly *de novo* emergence of genes, and contracting via gene loss [6,18–20]. Beyond the comparative genomic observations, estimates of the genome dynamics rates in prokaryotes have been obtained via explicit evolutionary reconstruction using maximum parsimony or maximum likelihood (ML) methods. These approaches typically employ the pattern of gene presence/absence in a set of species, which is mapped onto a guide phylogenetic tree [19–23]. All evolutionary reconstructions performed with widely different groups of bacteria and archaea infer various combinations of vertical inheritance, gene loss and gain. Averaged over long spans of evolution, gene loss appears to be a more common process than gene gain as shown for different groups of bacteria and archaea [19,21,23–25]. In obligate intracellular parasitic bacteria as well as in parasitic archaea, gene loss is the single dominant evolutionary process [7,26–28]. However, genome reduction, often described as streamlining, also prevails in the evolution of bacterial saprophytes [24,29] and some free-living microbes, particularly in marine environments [23,25,30,31]. These findings are compatible with the observation of the overall deletion bias in the evolution of prokaryotic (as well as eukaryotic) genomes [32,33]. Furthermore, gene loss has been reported to be a more uniform, ‘clock-like’ process than gene gain, which shows a stronger episodic character [19,29]. Taken together, these findings have inspired the concept of genome reduction as the ‘default’ evolutionary process counterbalanced by episodes of gene gain, primarily via HGT [34].

The discovery of the diversity of the gene repertoires, even among bacteria and archaea with closely related nucleotide sequences, led to a conceptual shift in microbiology. Under the new view of the microbial world, the key unit of microbial evolution is not the genome of an individual bacterium or archaeon but rather the pangenome of a prokaryote species [17,35–38]. The term ‘pangenome’ has been used alternatively to describe either the superset of the genes present in the genomes of all sequenced isolates of a given species, or the entire pool of genes that are potentially available for acquisition to the given species (or an otherwise defined monophyletic group of genomes) over the course of its evolution. Hereinafter, to avoid ambiguity, we restrict the use of ‘pangenome’ to denote the empirically detected superset of genes and use the term ‘supergenome’ [39] to refer to the entire gene reservoir. Obviously, the supergenome of any microbial species cannot be characterized directly and can only be estimated from the analysis of samples of the relevant genomes. Such estimates have pointed to vast, ‘open’ supergenomes for most prokaryotes because analysis of newly sequenced isolates did not show any signs of saturation of new gene discovery [36,37,40]. However, for a minority of bacteria, the supergenomes appear to be ‘closed’, with new genomes adding few if any new genes [36,37,40]. Attempts to estimate microbial supergenome sizes have been made using either statistical approaches or explicit mathematical models of the evolutionary process. In particular, Snipen *et al*. [41] estimated the supergenome size for several bacteria using a binomial mixture approach [42] to approximate the gene frequency distribution in an analyzed set of genomes. This analysis, unlike the earlier approximations, yielded closed and relatively small supergenomes that were only several fold larger than a typical microbial genome. A recent model of microbial (pan)genome evolution by gene replacement, known as the Infinitely Many Genes model, under which the replacing genes are drawn from a formally infinite reservoir [43,44], also suggested a close but much larger supergenome for the cyanobacterium *Prochlorococcus*. On the whole, the accuracy of the available supergenome estimates and the validity of the underlying models remain uncertain. Thus, delineation of supergenomes across the diversity of bacteria and archaea and elucidation of the factors that underlie the supergenome evolution are major tasks for evolutionary microbial genomics.

HGT is at present universally recognized as a major factor in the evolution of prokaryotes and a key source of innovation and adaptation to new environments and lifestyles [5,18,45–47]. However, attempts at quantification have yielded widely different estimates of the prevalence of HGT. Some early studies that involved a small number of genomes resulted in modest estimates, which implied a limited importance for HGT compared to vertical inheritance [21,48,49]. More recent phylogenomic analyses that included larger sets of genomes widely representative of the bacterial and archaeal diversity, generally reveal a much greater level of HGT [50–56]. For example, a quantitative assessment of the contributions of vertical inheritance and HGT to the evolution of prokaryotes based on the topological comparison of thousands of phylogenetic trees suggested that nearly two-thirds of evolutionary events originate from HGT [55]. Furthermore, evidence has been presented that HGT rather than gene duplication is the principal contributor to the evolution of gene families in prokaryotes [57].

We were interested in taking a comprehensive census of various events of genome dynamics across the diversity of prokaryotes. To obtain reliable rates of these events, we sought to analyze groups of multiple, closely related genomes so that robust identification of gene orthology and estimation of phylogeny and evolutionary distances would be ensured. The rates of gene dynamics were estimated for 35 clusters of prokaryotic genomes that make up an updated version of the collection of alignable tight genome clusters (ATGCs) [58] using a phylogenetic birth-and-death ML model [22,23]. The results reveal extremely rapid genome dynamics, albeit with broad ranges of gene loss and gain rates among prokaryotic species, and indicate an overall tendency to genome contraction, which is partially compensated by gene gain via HGT. We show that the overall flux of genes is the defining parameter of genome dynamics and provide estimates of the supergenome size for diverse groups of prokaryotes.

## Results

### Genome dynamics in prokaryotes: extensive gene family loss and gain dominate over family expansion and reduction

We employed an updated version of the ATGCs [58] to reconstruct the genome evolution for 34 groups of bacteria and one group of archaea (Additional file 1: Table S1 and Additional file 2: Figure S1). From the clusters of orthologous genes (COGs) that are associated with each ATGC, we derived the phyletic patterns (i.e. the patterns of presence/absence of gene families in each genome) as well as data on the number of members of each family including all paralogous genes. These patterns were mapped onto the phylogenetic tree of the respective ATGC (see Methods) and employed for the evolutionary reconstruction using Count, an ML method based on a phylogenetic gene birth-and-death model [22]. It should be emphasized that the COGs derived under this procedure account for the entire pangenome of each ATGC, and thus include genes shared between any number of organisms within the ATGC as well as genes unique to a single genome (singletons). Thus, no biases that could result from using a subset of genes preselected on certain criteria, such as, for example, the degree of sequence conservation, affect the estimates described below.

The rates of four types of elementary evolutionary event (hereinafter called genome dynamics events or GDEs) were analyzed: (i) gain of a gene family not present in the ancestor node (hereinafter, gain, for brevity), (ii) loss of all gene family members (loss), (iii) expansion of a gene family, i.e. addition of one or several family members (expansion) and (iv) reduction of a gene family, i.e. elimination of one or several family members (reduction). In mechanistic terms, gains are most likely to originate from HGT, and perhaps on rare occasions, from *de novo* gene birth; extreme divergence of duplicated genes that could lead to the appearance of a new family is highly unlikely on the short evolutionary scale of an ATGC. Gene family expansion is a combination of bona fide gene duplication and acquisition of a new member of a pre-existing family via HGT (here we do not attempt to distinguish between these two sources).

**Figure 1:**
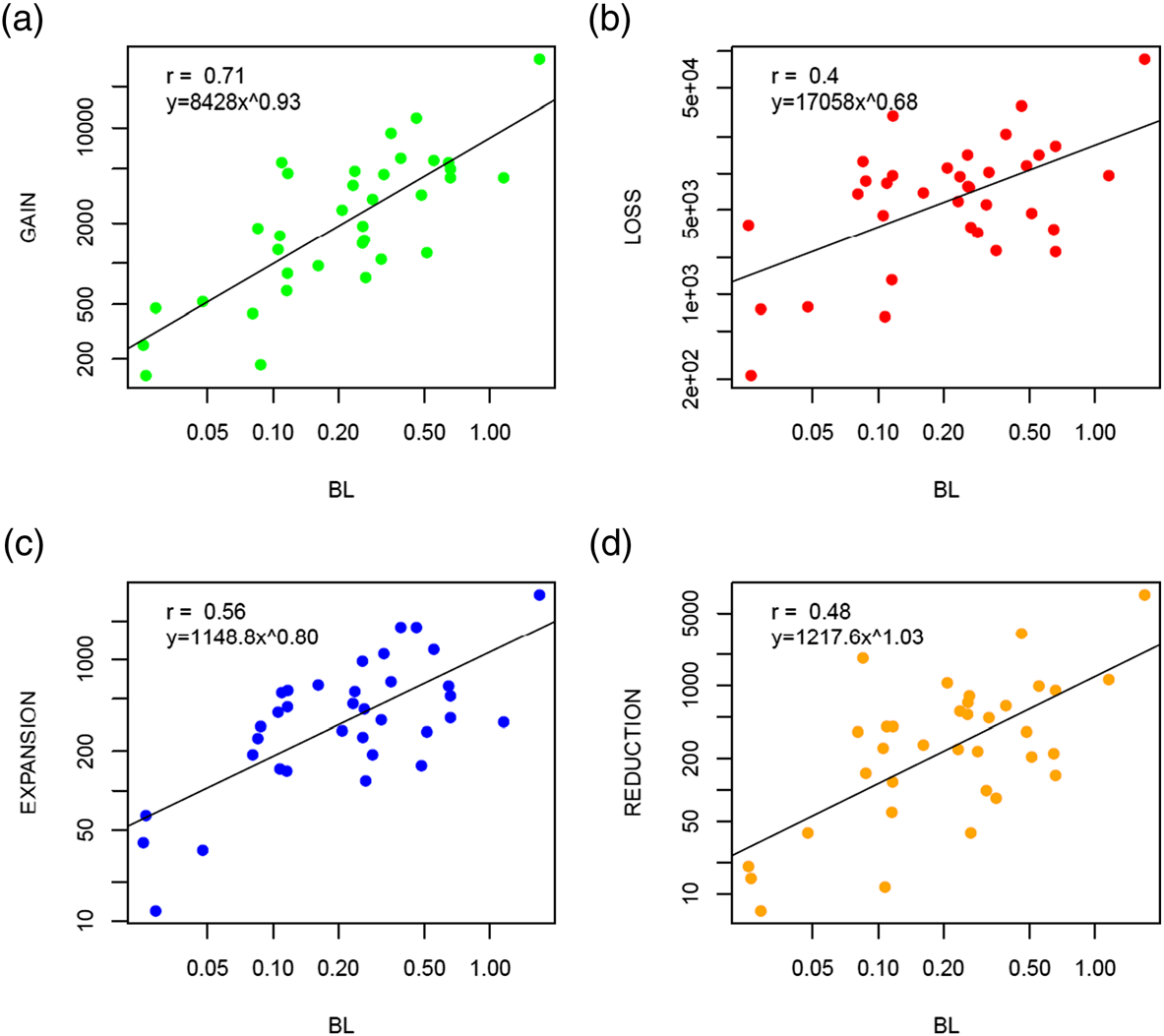
**The clock of genome dynamics.** The figure shows the correlation of branch lengths and number of **(a)** gains, **(b)** losses, **(c)** expansions and **(d)** reductions. It excludes singletons, i.e., gains in the terminal branches of the tree. Both *x* and *y* axes are have a logarithmic scale. All *P* < 0.0001. BL, branch length or number of nucleotide substitutions per site.

The number of GDEs of each type associated with each tree branch shows a strong significant positive correlation with the branch length (Figure 1, Additional file 2: Figure S2 and Additional file 3: Table S2). Thus, all these events appear to occur under a genomic clock, by analogy to the traditional molecular clock of sequence evolution [59]. The accuracy of the genomic clock was found to be the highest for gene family gain and the lowest for gene family loss (Figure 1) although this difference has to be taken with caution due to the limited number of data points (ATGCs). A bootstrap analysis (1,000 replicates) of the GDE rates shows that the estimated rates are not disproportionately affected by a small number of outliers and also supports the observation on the wider scatter of the loss rate compared to the gain rate (Additional file 2: Figure S3). Previous analyses performed on genomes representing diverse branches of bacteria, have suggested that gene loss was more of a clock-like process than gene gain, which showed a tendency to occur in isolated episodes [19]. The present results suggest that this trend is not manifest at the short evolutionary scale of the ATGCs, compatible with more recent observations of an apparent clock-like character of HGT, at least among universally conserved prokaryotic genes [60].

The demonstration of the existence of the genomic clock justifies the estimation of the rates of gain, loss, expansion and reduction per unit of nucleotide substitution and in what follows, we primarily use this measure.

Table 1 shows the rates of each type of GDE for the 35 ATGCs. Five major trends are immediately apparent:

**Table 1:** **Rates of the four types of genome dynamics events** ^**a**^

**ATGC**	**Genera**	**Gain**		**Loss**		**Expansion**		**Reduction**	
		**Site**	**Gene**	**Site**	**Gene**	**Site**	**Gene**	**Site**	**Gene**
ATGC001	*Enterobacteria*	18563	17.3	50517	47.1	1864	1.7	4405	4.1
ATGC002	*Enterobacter–Klebsiella*	25866	24.5	6448	6.1	1914	1.8	237	0.2
ATGC003	*Streptococcus*	11894	11.8	53500	53.0	1368	1.4	5092	5.0
ATGC004	*Streptococcus*	5633	5.5	29233	28.5	1601	1.6	2984	2.9
ATGC005	*Streptococcus*	11940	11.3	42215	40.0	3755	3.6	2380	2.3
ATGC014	*Bacillus*	25485	25.5	77488	77.7	3895	3.9	6908	6.9
ATGC015	*Bacillus*	6521	6.6	25293	25.5	804	0.8	1343	1.4
ATGC021	*Chlamydia*	16346	14.0	25968	22.3	419	0.4	240	0.2
ATGC022	*Chlamydia–Chlamydophila*	5434	5.2	11202	10.8	1213	1.2	524	0.5
ATGC025	*Mycobacterium*	50823	45.9	74868	67.6	5109	4.6	3708	3.3
ATGC033	*Mycoplasma*	5865	4.6	8335	6.6	2484	2.0	548	0.4
ATGC046	*Rickettsia*	6677	7.4	23674	26.4	325	0.4	750	0.8
ATGC052	*Helicobacter*	3733	3.6	8293	8.0	288	0.3	974	0.9
ATGC054	*Staphylococcus*	21512	19.9	146937	135.8	2924	2.7	21696	20.1
ATGC056	*Lactobacillus*	3417	3.3	17553	17.2	1094	1.1	314	0.3
ATGC067	*Corynebacterium*	10013	9.0	146236	131.5	1582	1.4	734	0.7
ATGC068	*Corynebacterium*	5338	4.9	82891	76.0	2335	2.1	4414	4.0
ATGC072	*Pseudomonas*	10467	9.2	25516	22.5	2162	1.9	1766	1.6
ATGC082	*Clostridium*	7223	6.6	82676	75.8	3745	3.4	1030	0.9
ATGC089	*Burkholderia*	39593	35.5	252406	226.3	4988	4.5	3417	3.1
ATGC090	*Burkholderia*	15276	13.6	54192	48.2	4582	4.1	1644	1.5
ATGC094	*Sulfolobus*	5956	6.1	42639	43.4	3931	4.0	1643	1.7
ATGC105	*Bifidobacterium*	10383	8.3	11148	9.0	655	0.5	817	0.7
ATGC106	*Bifidobacterium*	11039	8.9	16473	13.3	742	0.6	812	0.7
ATGC109	*Listeria*	7650	7.5	3411	3.4	551	0.5	207	0.2
ATGC121	*Shewanella*	8576	7.3	5340	4.5	973	0.8	348	0.3
ATGC128	*Yersinia*	20234	17.5	39460	34.2	2391	2.1	2369	2.1
ATGC135	*Xanthomonas*	13993	12.2	31495	27.4	3413	3.0	1518	1.3
ATGC137	*Brucella–Ochrobactrum*	16268	15.6	25177	24.1	1968	1.9	1033	1.0
ATGC138	*Neisseria*	7278	6.4	29817	26.4	990	0.9	2638	2.3
ATGC139	*Francisella*	2324	2.0	9075	7.7	546	0.5	401	0.3
ATGC144	*Campylobacter*	14997	14.6	6085	5.9	1365	1.3	109	0.1
ATGC153	*Acinetobacter*	5416	5.1	54454	51.7	3747	3.6	2029	1.9
ATGC163	*Propionibacterium*	2948	2.7	13223	12.0	446	0.4	145	0.1
ATGC186	*Legionella*	2082	1.9	99232	89.9	3546	3.2	1647	1.5
Median		10013	8.3	29233	26.4	1864	1.7	1343	1.3

^a^For each ATGC, the rates of each type of GDE per nucleotide substitution per site and per nucleotide substitution per gene are indicated.

**Figure 2:**
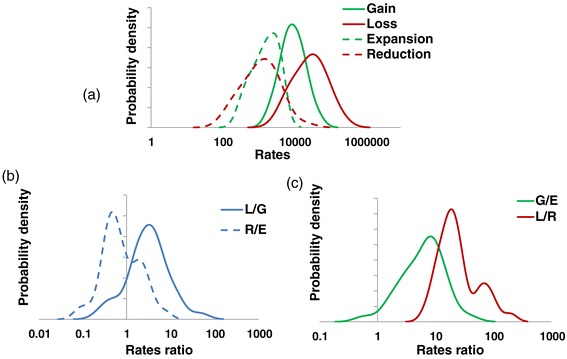
**Distributions of the genome dynamics rates across the ATGCs. (a)** Rates of gain, loss, expansion and reduction per nucleotide substitution per site. **(b)** Loss/gain and reduction/expansion ratios. **(c)** Gain/expansion and loss/reduction ratios. G/E, gain/expansion; L/G, loss/gain; L/R, loss/reduction; R/E, reduction/expansion.

i). The rates of gain and loss are approximately an order of magnitude greater than the rates of expansion and reduction.
ii). The loss rate typically is nearly threefold higher than the gain rate.
iii). The expansion rate is almost 1.5 times higher than the reduction rate.
iv). The rates of all types of GDE vary within a broad range, spanning almost two orders of magnitude (Figure 2a); the ratios between the rates of different events vary within similar ranges (Figure 2b,c). This trend is independent of the number of species in an ATGC (Additional file 2: Figure S4).
v). The rates of genome change are remarkably high, typically tens of thousands of GDEs per nucleotide substitution per site, or tens to hundreds of GDEs per substitution per gene (Table 1, Additional file 3: Table S2).

**Figure 3:**
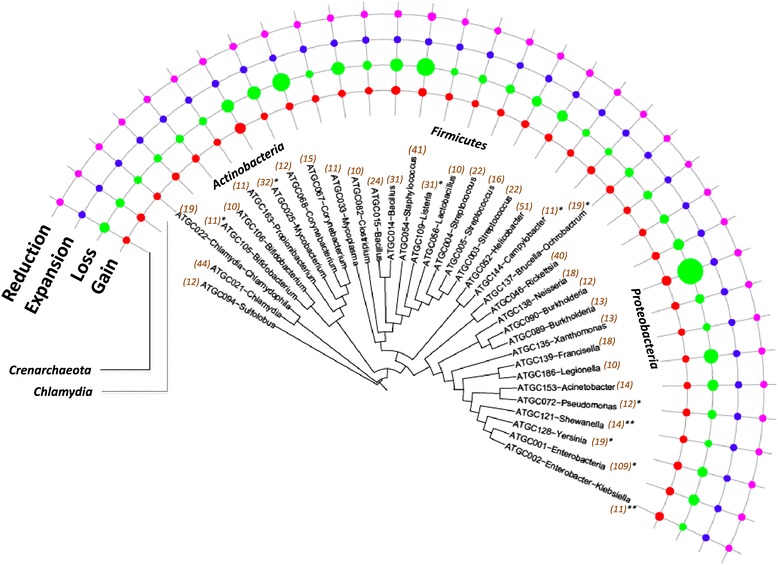
**Distribution of the gain, loss, expansion and reduction rates over the evolutionary tree of prokaryotes.** The tree is from MicrobesOnline [62]. The areas of the circles are proportional to the rates of the respective events to a logarithmic scale. The numbers in parenthesis indicate the number of species in the ATGC. The ATGCs with episodes of rapid gene gain are denoted with *(<10% of branches) or **(>10% of branches). ATGC, alignable tight genome cluster.

On the whole, the dominant process in prokaryote genome evolution is the loss of gene families, i.e. genome contraction. This finding provides definitive quantitative support for the previous, more qualitative conclusions of the importance of genome streamlining in evolution, particularly among prokaryotes [19,21,23,25,34,61]. However, due to the high variation in the rates of different processes of genome evolution, this general trend is reversed in some of the analyzed groups of microbes (Table 1). In particular, despite the overall dominance of gene family loss, there are clear gainers among the analyzed bacteria, such as *Enterobacter*, *Klebsiella*, *Campylobacter* and *Listeria*. Furthermore, the switch from the loss mode of evolution to the gain mode appears to occur in the course of evolution of some relatively close-knit groups of bacteria (Table 1 and Figure 3). Specifically, the Enterobacteriaceae and Campylobacterales clades include both gainer and loser ATGCs (Table 1 and Figure 3). Rapid, extensive gene loss (loss + reduction to gain + expansion ratio >10) is particularly prominent in *Legionella* and *Corynebacterium* (Table 1 and Figure 3).

### Estimates of gene dynamics rates and phylogenetic depth

**Figure 4:**
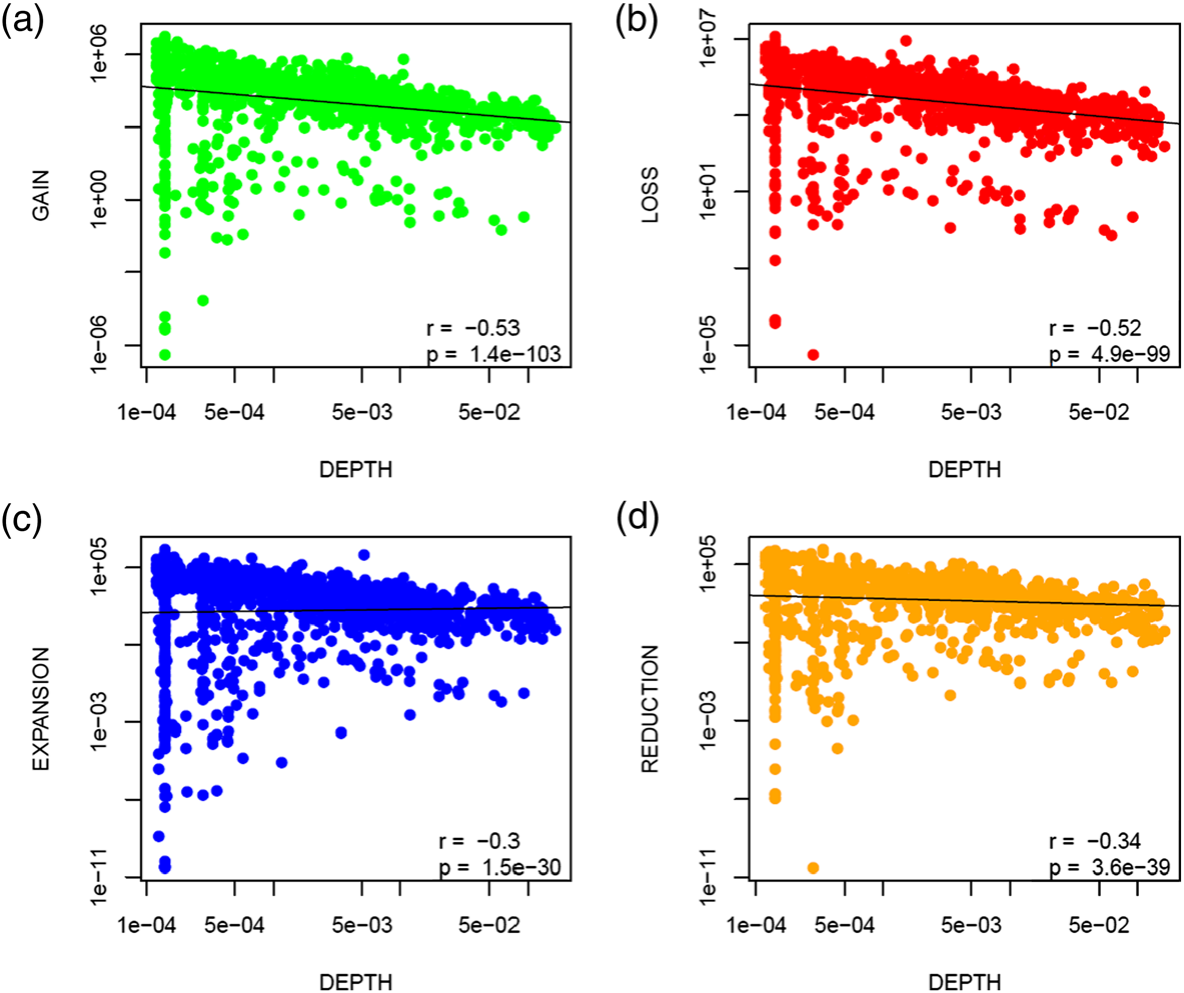
**Dependence of the rates of gains, losses, expansion and reductions on phylogenetic depth. (a)** Gains, **(b)** losses, **(c)** expansions and **(d)** reductions per unit of branch length vs the phylogenetic depth. The figure excludes singletons, i.e., gains in the terminal branches of the tree are not represented. Both *x* and *y* axes have a logarithmic scale. The phylogenetic depth is measured in the number of nucleotide substitutions per site.

When the events are analyzed on individual tree branches, the rates of all four types of GDE strongly and negatively correlate with the phylogenetic depth of the respective branch (Figure 4). Most likely, this observation reflects the fact that Count only estimates the number of GDEs for those gene families that survived in at least one extant genome. Genes that were present at some point during the history of the ATGC but have been subsequently lost, are missed altogether so that the corresponding GDEs do not contribute to the calculations. These findings point to the high prevalence of such transient GDEs in microbial evolution and suggest that our reported estimates (Table 1) represent the low bound of the actual gene flux.

To estimate the extent to which Count underreports the number of GDEs, we used the dependence of the estimated rates on the depth of the branch mid-point. Within each ATGC, the observed rates were normalized to 100% at the depth of 0.0001 substitutions per site (Figure 4; Additional file 2: Figure S5). The results indicate that at a phylogenetic depth of 0.1 (the deepest branches among all ATGCs), Count might underestimate the rates by up to 40%. At the more typical tree branch depth, the expected deficit is much lower. Thus, we expect our estimates to be accurate within a factor of 2 at most. Furthermore, we show that the relative GDE rates are consistent independent of the phylogenetic depth (Additional file 2: Figure S6). In agreement with the trend observed for individual branches, the ATGC-wide estimate of the gene flux rate (overall GDE rate; see below) also shows significant negative correlation with the total phylogenetic tree depth, estimated as the mean root-to-leaves distance (Additional file 2: Figure S7).

### Factors of microbial genome dynamics

**Figure 5:**
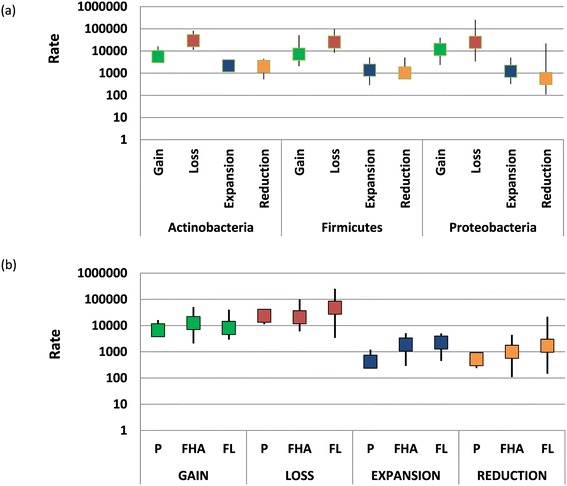
**Dependence of the rates of gain, loss, expansion and reduction on bacterial taxonomy and lifestyle. (a)** Rates of the four types of event for Actinobacteria, Firmicutes and Proteobacteria. **(b)** Rates of the four types of event for bacteria and archaea with three different lifestyles. FHA, facultative host-associated; FL, free-living; P, obligate intracellular parasite.

Despite the substantial variability among individual ATGCs, the genome dynamics rates appeared to be (nearly) randomly scattered across the diversity of prokaryotes (Figure 3) and in particular showed no significant differences between the three major bacterial phyla represented by multiple ATGCs, namely Proteobacteria, Actinobacteria, and Firmicutes (Figure 5a). Thus, the trends of genome contraction (loss and reduction) and expansion (gain and expansion) appear to hold for most of the lineages across the entire bacterial domain.

We also compared the rates of gain, loss, expansion and reduction between microbes with three lifestyles, free-living, facultative host-associated and obligate intracellular parasite (Figure 5b). Perhaps unexpectedly, given the typically much smaller genomes of the intracellular parasites, the overall relationship between the rates of the four types of event did not depend on the lifestyle: Loss > Gain > > Expansion > Reduction. Nevertheless, among the three groups, free-living bacteria present the highest rates of gain (not significant), expansion (*P* < 0.01 compared to parasites) and reduction (*P* < 0.01 compared to parasites), whereas obligate intracellular parasites and facultative host-associated bacteria show a modest but significantly higher rate of gene family loss than free-living bacteria (*P* < 0.05) (Figure 5b). Thus, on the whole, genomes of free-living prokaryotes appear to be more dynamic than genomes of intracellular parasites which is compatible with the greater exposure to HGT in extracellular compared to intracellular habitats.

Perhaps surprisingly, no connection was found to exist between the rates of the GDEs and the strength of the selection pressure on protein sequences estimated as the ratio of non-synonymous to synonymous substitution rates in protein-coding genes (*dN/dS*) [63], which shows a robust correlation with various ATGC-wide characteristics [64] (Additional file 2: Figure S8). Neither did we detect any dependence of the four GDE rates on the genomic GC content (Additional file 2: Figure S9), notwithstanding the strong positive correlation between the GC content and genome size [65,66], or with the genome shuffling rate (see Additional file 2: Figure S10 and Methods for the details of the shuffling rate calculation).

### Flux and balance in prokaryotic genome evolution

**Figure 6:**
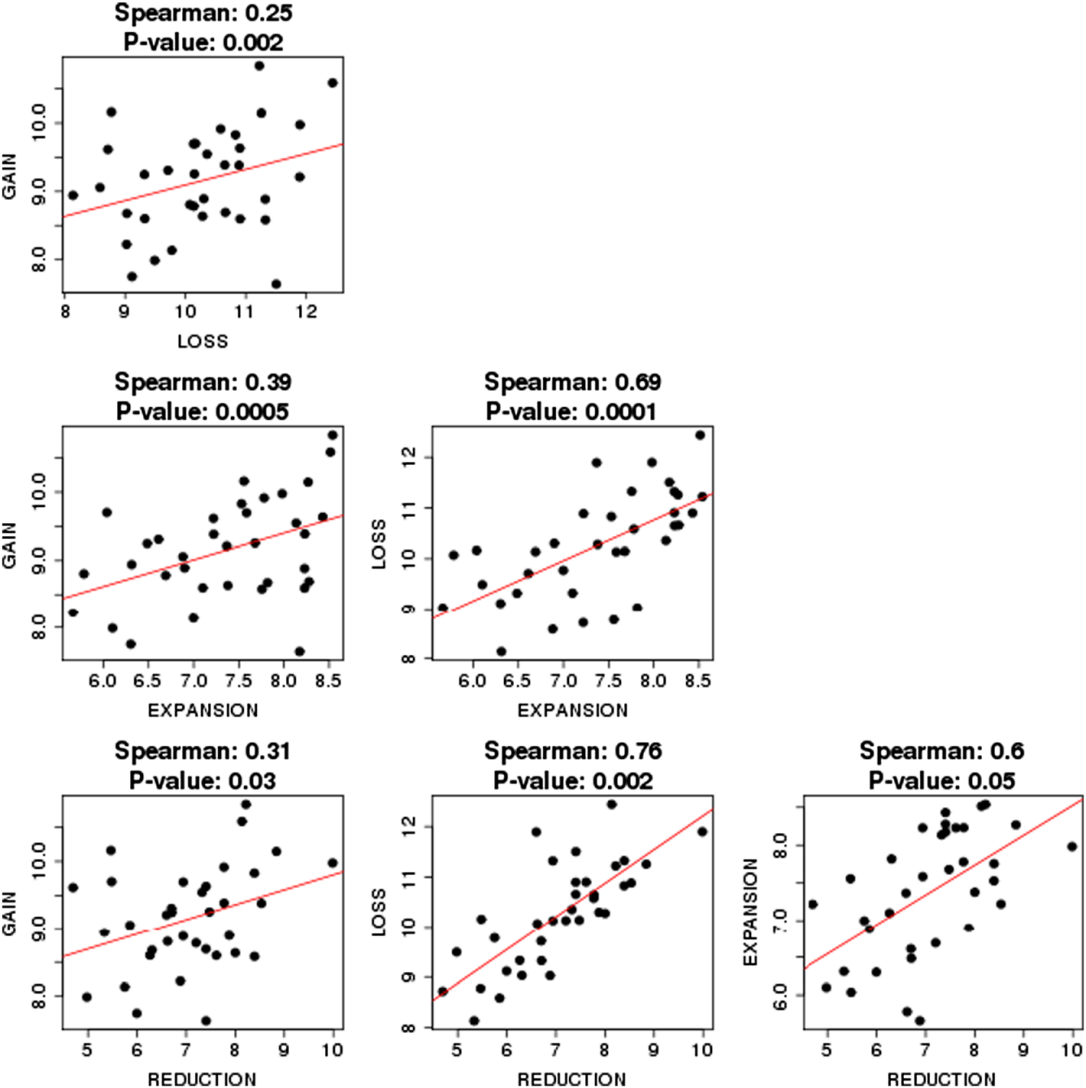
**Correlations between the rates of gain, loss, expansion and reduction.**

We further examined possible correlations between different types of GDEs. Strikingly, relatively high, statistically significant, positive correlation was shown to exist between all types of event (Figure 6). These findings suggest that the dynamics of genome evolution in prokaryotes is largely determined by the overall gene flux.

**Figure 7:**
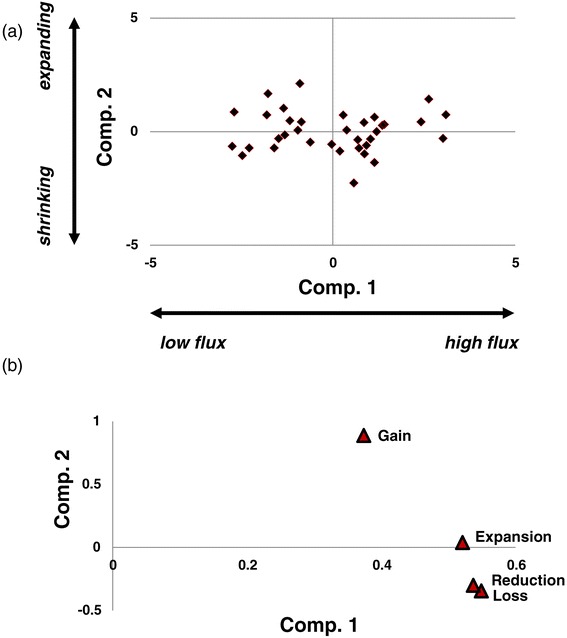
**Principal component analysis of the rates of gains, losses, expansions and reductions.** **(a)** XY-plot of the two first two principal components. **(b)** Principal component analysis loadings. Comp., component.

To further investigate key factors of genome dynamics, we performed principal component analysis (PCA) of the rates of gain, loss, expansion and reduction (Figure 7a and Additional file 2: Figure S11). In this case, the PCA was remarkably efficient in revealing major trends of genome evolution. The first principal component explained approximately 64% and the second principal component approximately 19% of the variance in the GDE rates, indicating that each of these composite variables reflected a major trend of genome evolution. The loadings plot (Figure 7b) shows that all four rates (gain, loss, expansion and reduction) contributed to the first principal component with the same sign. Accordingly, this principal component appears to reflect the overall gene flux, which thus appears to be the key determinant of genome dynamics. The second principal component was dominated by gene family gain and loss, which contribute with opposite signs (Figure 7b). Thus, this component reflects the balance of family loss and gain.

To account for the difference in the contributions of different GDEs to the net extent of the genome change (Loss > Gain > > Expansion > Reduction), we use the sum of all event rates as the measure of the total gene flux and the gain + expansion to loss + reduction ratio as the measure of the balance.

**Figure 8:**
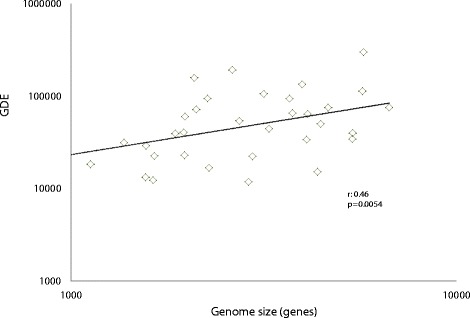
**Correlation between gene flux and genome size.** The horizontal axis shows the median number of genes in a genome in an ATGC. ATGC, alignable tight genome cluster; GDE, total gene flux (number of genome dynamics events per nucleotide substitution per site).

Gene flux but not the balance of the GDE positively and significantly correlates with the genome size (Figure 8 and Additional file 2: Figure S12). Combined with the observations on the transient character of many genomic events, this finding implies that the larger microbial genomes are products of recent and conceivably short-lived gene accretion.

### Evolutionary dynamics of different functional classes of genes

**Figure 9:**
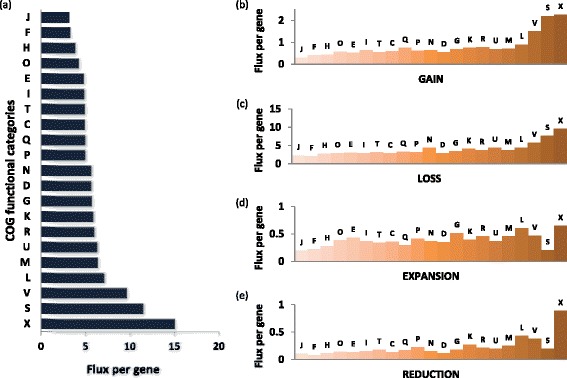
**Genome flux by COG functional categories. (a)** Flux. **(b)** Gain. **(c)** Loss. **(d)** Expansion. **(e)** Reduction. Designations of the functional categories (modified from [67]): C, energy production and conversion; D, cell division; E, amino acid metabolism and transport; F, nucleotide metabolism and transport; G, carbohydrate metabolism and transport; H, coenzyme metabolism; I, lipid metabolism; J, translation; K, transcription; L, replication and repair; M, membrane and cell wall structure and biogenesis; N, secretion and motility; O, post-translational modification, protein turnover and chaperone functions; P, inorganic ion transport and metabolism; Q, biosynthesis, transport and catabolism of secondary metabolites; R, general functional prediction only (typically, prediction of biochemical activity); S, function unknown; T, signal transduction; U, intracellular trafficking and secretion; V, defense systems; X, mobilome. COG, cluster of orthologous genes.

We further estimated the overall gene flux and the rates of each type of GDE for broad functional categories of genes as defined in the COGs [67,68]. All rates showed largely consistent ranking of functional categories (Figure 9), in agreement with the overall positive correlation between them (Figure 6). Predictably, genes encoding protein components of the translation system, which constitute the great majority of the (nearly) universal genes in cellular life forms [69–71], make up the category with the lowest flux per gene (mostly static), closely followed by enzymes of nucleotide and coenzyme metabolism as well as molecular chaperones, which also tend to be highly conserved in evolution (Figure 9). In contrast, by far the most dynamic class included genes of mobile elements, followed by uncharacterized genes and genes involved in defense functions (Figure 9). It appears likely that numerous genes in the uncharacterized category actually are unidentified components of the mobilome or defense systems [72], suggesting that, as one might expect, these two categories jointly make up the most dynamic component of microbial genomes. The difference between the per gene flux rates of the mobilome components and the translation genes was approximately fourfold, and when the gain rates were compared, the difference was greater than an order of magnitude. These findings are generally compatible with the patterns of long-term gene conservation [6] and emphasize the heterogeneity of gene dynamics in bacterial and archaeal genomes.

### Supergenome size estimation

The results described above indicate that prokaryote genome evolution is an extremely dynamic process that involves rapid gain and loss of numerous gene families. This process can be naturally represented as sampling of a gene pool by the evolving genomes, which draw new gene families at random. We denote this gene pool the supergenome of an ATGC, to differentiate it from the pangenome, the empirically observed superset of genes of a group of genomes. The size of the supergenome is unknown but can be estimated from the number of families that have been gained multiple times: obviously, with a vast supergenome, the chance to draw the same family again is effectively nil, whereas with a supergenome only slightly exceeding the typical genome size of a given group, many families will be gained repeatedly. We developed an ML model to estimate the size of the supergenome from the number of repeated gains; the estimates were obtained for two models of genome evolution, namely the simplest conceivable model, with a uniform probability of drawing a gene from the supergenome and a more complex model with a power-law distribution of the drawing probabilities (see Methods for details).

**Figure 10:**
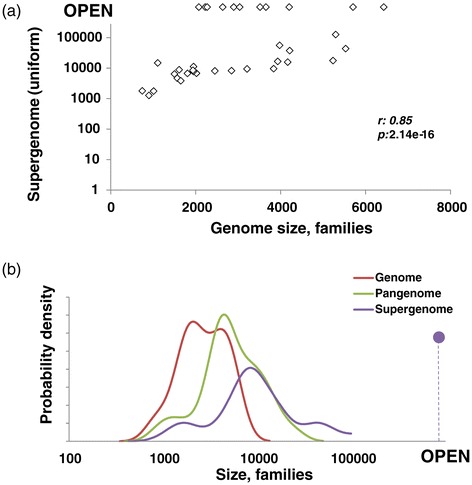
**Comparison of genome, pangenome and estimated supergenome sizes. (a)** Median genome vs supergenome size. **(b)** Density distribution of median genome, pangenome and supergenome size.

**Figure 11:**
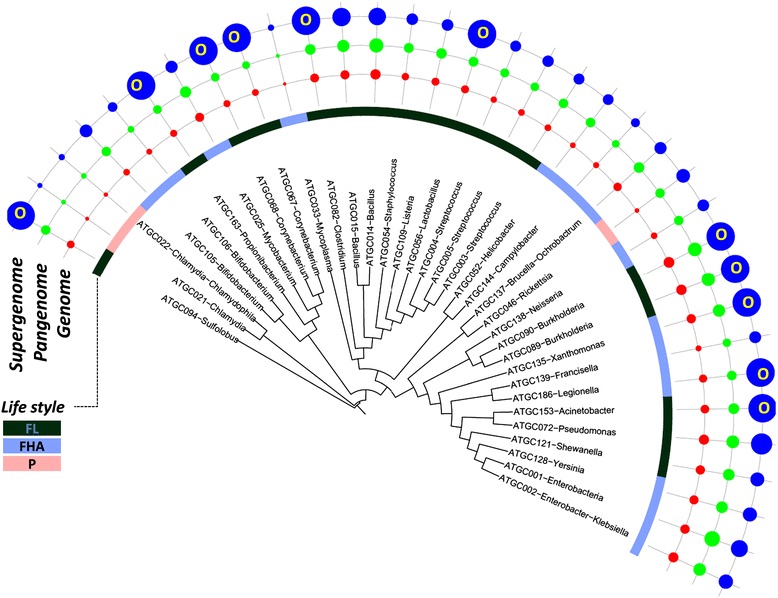
**Distribution of the median genome, pangenome and estimated supergenome sizes over the evolutionary tree of prokaryotes.** The tree is from MicrobesOnline [73]. Areas of the circles are proportional to the number of genes in the respective genomes (median), pangenome, a006Ed supergenome. FHA, facultative host-associated; FL, free-living; O, open supergenome; P, obligate intracellular parasite.

The results obtained with the two approaches were consistent and showed a wide spread of estimated supergenome sizes, from approximately four genomic equivalents (hence numerous repeated gains) to effectively open supergenomes (no or very few repeated gains) (Table 2 and Figures 10a and 11). In ATGCs with closed supergenomes (Table 2), the characteristic size of the supergenome was estimated at about an order of magnitude larger than the typical number of families in a genome (Figure 10b). For these closed supergenomes, the estimates were highly reliable, with the confidence intervals typically less than 10% of the estimate (Table 2).

**Table 2:** **Supergenome size estimates**

**ATGC**	**Genera**	***F***	***P***	***K***	***M***	***S*** **, uniform**		***S*** **, power**	
						***a***	***b***	***a***	***b***
ATGC001	*Enterobacteria*	4215	24845	38293	13448	37267 ± 458	8.8	45092	10.7
ATGC002	*Enterobacter–Klebsiella*	5234	10802	13454	2652	17560 ± 429	3.4	18403	3.5
ATGC003	*Streptococcus*	2020	4478	5330	852	6754 ± 297	3.3	6754	3.3
ATGC004	*Streptococcus*	1808	3746	4132	386	6664 ± 554	3.7	6664	3.7
ATGC005	*Streptococcus*	1948	3466	3692	226	7669 ± 877	3.9	7669	3.9
ATGC014	*Bacillus*	5533	16678	19554	2876	43302 ± 1676	7.8	43303	7.8
ATGC015	*Bacillus*	3976	9242	9871	629	55655 ± 7699	14.0	78848	19.8
ATGC021	*Chlamydia*	901	1168	1395	227	1260 ± 31	1.4	1344	1.5
ATGC022	*Chlamydia–Chlamydophila*	1011	1441	1699	258	1758 ± 83	1.7	1759	1.7
ATGC025	*Mycobacterium*	3836	7293	9481	2188	9484 ± 190	2.5	13514	3.5
ATGC033	*Mycoplasma*	742	883	899	16	1784 ± 539	2.4	1785	2.4
ATGC046	*Rickettsia*	1107	4454	5050	596	14677 ± 1293	13.3	24011	21.7
ATGC052	*Helicobacter*	1501	4568	6270	1702	6377 ± 179	4.2	7246	4.8
ATGC054	*Staphylococcus*	2452	4815	5413	598	8071 ± 464	3.3	8322	3.4
ATGC056	*Lactobacillus*	2896	4893	4896	3	Open		Open	
ATGC067	*Corynebacterium*	2074	2721	2721	0	Open		Open	
ATGC068	*Corynebacterium*	2227	3453	3460	7	Open		Open	
ATGC072	*Pseudomonas*	5297	11389	12037	648	125699 ± 20767	23.7	125700	23.7
ATGC082	*Clostridium*	3655	5993	5993	0	Open		Open	
ATGC089	*Burkholderia*	5707	13381	13546	165	Open		Open	
ATGC090	*Burkholderia*	6428	14540	14659	119	Open		Open	
ATGC094	*Sulfolobus*	2638	4471	4479	8	Open		Open	
ATGC105	*Bifidobacterium*	1952	4352	4986	634	11174 ± 829	5.7	11174	5.7
ATGC106	*Bifidobacterium*	1568	2018	2087	69	4631 ± 1020	3.0	5619	3.6
ATGC109	*Listeria*	2843	5834	7588	1754	8207 ± 210	2.9	11712	4.1
ATGC121	*Shewanella*	4166	8090	9427	1337	15597 ± 633	3.7	15929	3.8
ATGC128	*Yersinia*	3934	8287	9406	1119	16479 ± 778	4.2	20747	5.3
ATGC135	*Xanthomonas*	4204	9814	9876	62	Open		Open	
ATGC137	*Brucella–Ochrobactrum*	3212	6012	7213	1201	9376 ± 343	2.9	11086	3.5
ATGC138	*Neisseria*	1937	4344	4840	496	8395 ± 670	4.3	8395	4.3
ATGC139	*Francisella*	1613	3247	3426	179	8801 ± 1360	5.5	8801	5.5
ATGC144	*Campylobacter*	1650	2637	3101	464	3811 ± 179	2.3	3811	2.3
ATGC153	*Acinetobacter*	3517	6455	6497	42	Open		Open	
ATGC163	*Propionibacterium*	2270	3485	3488	3	Open		Open	
ATGC186	*Legionella*	3038	4587	4587	0	Open		Open	

*a*, number of gene families in the estimated supergenome; *b*, supergenome size in genome units (ratio of the estimated number of families in the supergenome to the median number of families in a genome given by *F*); *F*, median number of gene families per genome; *K*, total number of gene family gains; *M*, number of multiple gene family gains; *P*, pangenome size (sum total of the gene families); *S*, supergenome size (estimated under the uniform and power law models; see text for details).

The estimated supergenome size positively correlated with the mean genome size in an ATGC, indicative of a trend of genome growth dependent on the pool of available genes (Figure 10a). By contrast, and perhaps unexpectedly, the estimated supergenome size, expressed either as the number of families or relative to the genome size (in genomic units), does not correlate significantly with the gene flux or with gain, loss, expansion and reduction rates separately (Additional file 2: Figure S13). In other words, microbes with large supergenomes can, at least transiently, evolve in a relatively static regime and conversely microbes with rapidly evolving genomes can have small supergenomes.

Supergenome size estimates show a strong positive correlation with the total tree depth (Additional file 2: Figure S14a). This dependence is likely to stem from at least two factors. First, the deeper the divergence of the ATGC, the more variation in the history of the environments, and therefore, in the adaptive requirements and the available gene pool, is expected. Second, our supergenome estimate procedure is intrinsically dependent on the number of multiple gene gains, derived from the phyletic patterns. As shown above (Figure 4a; Additional file 2: Figures S5), the gene gain rate is underestimated in the deeper trees and branches; accordingly, the number of multiple gene gains is underestimated as well, resulting in inflation of the supergenome size estimates.

Supergenome size estimates also show a strong and significant negative correlation with the ATGC-wide estimate of the *dN*/*dS* ratio (Additional file 2: Figure S14b). This dependence might reflect genuine relationships between the characteristic population dynamics of the respective group, which affect the strength of the purifying selection on the protein-coding genes [64]. However, the *dN*/*dS* ratio estimates themselves are negatively correlated with the ATGC tree depth (Additional file 2: Figure S14c) and genome size [64] (Additional file 2: Figure S14d). Thus, the apparent connection between the supergenome size and the protein-level selection might be due, at least in part, to indirect effects.

There are clear connections between the obtained supergenome size estimates and the microbial lifestyle. Thus, nine of the eighteen free-living microbes in the analyzed set but only two of the seventeen host-associated microbes were estimated to possess open supergenomes (chi-squared, *P* = 0.015) (Table 2 and Figure 11). This substantial excess of open supergenomes among free-living organisms could be expected as the result of their greater exposure to diverse gene pools. Also in line with the lifestyles of the respective microbes, by far the smallest supergenomes were estimated for intracellular (*Chlamydia*) and extracellular (*Mycoplasma*) parasites with highly reduced genomes; intracellular parasites with somewhat larger genomes (*Rickettsia*) appeared to have larger supergenomes, suggestive of a distinct evolutionary history (Table 2 and Figure 11).

Previously published supergenome size estimates (mostly referred to as pangenome size estimates by the authors) can be broadly classified into three categories. Estimates based on the sampling curve use an approximation for the number of new genes brought into the pangenome by additional genomes with a (semi-arbitrary) function [35–37,40]. If such a function converges to a finite total number of genes, a supergenome is considered closed and a quantitative estimate can be produced, otherwise the supergenome is considered open. Another category of estimates employs an explicit sampling model that assumes random independent sampling of genes from a common pool into individual genomes [41,42]. The third category employs a tree-based model of evolution of a group of genomes where sampling is performed along the tree branches [43,44]. The latter two approaches explicitly or implicitly fit the sampling model parameters to the observed distribution of gene frequencies in the analyzed set of genomes.

A comparison of the published estimates with those obtained in this work (Additional file 4: Table S3) shows that our estimates are consistently higher than those obtained with the models that assume that genomes are random independent collections of genes. The likely explanation is that the independence assumption inherent in these models leads to overestimates of the number of multiple gene gains by counting such gains for a family found in each genome within a clade, whereas the most likely scenario is that this family had been gained only once. Overestimation of the number of multiple gains necessarily leads to underestimation of the supergenome size. The estimates based on sampling curves yield open supergenomes for the majority of microbial groups. These approaches attempt to derive the exponent of the approximating power functions from the limited available samples, leading to much uncertainty. The fact that the infinitely many genes model could not be rejected, even in a tree-based analysis [44], might indicate that many microbial supergenomes are close to the closed/open boundary. Thus, it appears possible that neither the sampling curves nor the distributions of gene frequencies contain sufficient information to produce robust supergenome estimates. The approach employed here used the number of multiple gene gains directly inferred from the superposition of phyletic patterns of genes over a phylogenetic tree and, being independent of the assumptions of other models, could potentially improve the reliability of supergenome estimates, compatible with the narrow confidence intervals (Table 2).

## Discussion

### The rapid dynamics of prokaryotic genome evolution

The analysis of prokaryotic genome dynamics described here unequivocally shows that rapid gene flux involving extensive loss of genes and families, partially balanced by gain of new gene families via HGT, is the principal mode of microbial evolution. Indeed, the estimated rates of gene family gain and loss in some groups of bacteria are such that multiple genes appear to come and go over the time required for a single nucleotide substitution to occur in an evolving gene. These findings are compatible with experimental results demonstrating bacterial genome contraction in real time [73].

Given that the great majority (typically, around 90%) of nucleotide substitutions in evolving microbial genomes are silent [64,74], and even among those that affect protein sequences many are effectively neutral, it seems indisputable that rapid gene flux is the most important route of change in prokaryotic evolution. Notably, the present results show substantially greater rates of gene family loss and gain compared to family contraction and expansion rates. Thus, the gene flux is not only rapid and extensive but often leads to qualitative changes in the gene repertoires. In general terms, these results emphasize that prokaryotic evolution is largely driven not by small variations, such as single nucleotide substitutions, but by much more dramatic changes brought about by HGT and gene loss.

In agreement with previous observations made for larger evolutionary scales [19,21], we found that on the microevolutionary perspective that is provided by the ATGC analysis, gene family loss prevails over gain, and the difference between the rates of loss and gain is often substantial. Why, then, do prokaryotic genomes not shrink out of existence? The answer is likely to be twofold. Some of the bacteria actually might be headed towards extinction as observed for the tiny genomes of some intracellular parasites [7,26,27]. However, the more common scenario would involve evolving prokaryotic lineages going through long phases of genome contraction, and our analysis caught most of them, punctuated by shorter bursts of extensive gene gain, which we detected in a few groups and which compensate for the gradual gene loss. The non-clock like character of gene gain inferred on a longer timescale [19] implies that such periods could be short but would involve massive amounts of genetic material, making detection of such episodes through comparative analysis of tight groups of microbes unlikely.

The median relative and to a large extent even absolute (per nucleotide substitution) rates of loss, gain, reduction and expansion are highly consistent between the major bacterial phyla, surprisingly do not depend on the ATGC-wide *dN*/*dS* estimates, and only weakly depend on the lifestyle (parasitic vs independent). However, these rates showed a clear link to gene function, in a good agreement with prediction made from the long-term conservation of different functional classes of prokaryotic genes.

The variation in the GDE rates among individual ATGCs is substantial, spanning nearly two orders of magnitude (Table 1). At present, it is unclear why some genomes rotate genes fast and others slowly, apparently irrespective of the size of the gene pools that are available for HGT. The weakness of taxonomic coherence and connection to the microbial lifestyle imply that the defining factors have to do with specific, local aspects of microbial ecology. Identification of these factors is a major challenge for future comparative genomic and experimental studies.

A limitation of the evolutionary reconstructions reported here is that the ML approach implemented in Count [22,75] takes as input the gene family membership matrix, without explicitly exploiting information on the level of sequence similarity and phylogenetic relationships within individual families. In principle, more precise reconstructions taking into account this additional information are possible through the use of tree reconciliation approaches that compare gene trees to species trees [76–78]. Most of the available tree reconciliation algorithms are computationally prohibitive but recently, efficient, fast methods have been reported [79,80]. Nevertheless, application of even this promising approach on the scale addressed in this work and statistical assessment of the results remain challenges for future studies. In practice, it appears that tree reconciliation has the potential to uncover cases where a gene in a particular lineage, although included in a COG, shows a phylogenetic position significantly different from that in the species tree, and hence could actually have been acquired via HGT. Such cryptic HGT events could be of two kinds: (i) displacement of an existing family member by a xenolog, i.e. a homolog from a distant lineage, known as xenologous gene displacement and (ii) acquisition of a ‘pseudoparalog’ , i.e. an additional family member, again from a distant source [18]. Importantly, only events within families would be involved and the family gain estimates would remain unaffected. The findings of this work indicate that the contribution of pseudoparalog acquisition is small, given that the estimated family expansion rates are about an order of magnitude lower than the gain rates. The extent of xenologous gene displacement is unknown and remains an interesting target for further analysis. Regardless, it should be emphasized that the refinement of the GDE rate estimates that potentially could be obtained through tree reconciliation, can only lead to an upward reassessment of the rate of HGT. Thus, taking into account also the apparent underestimation by Count of events at deeper branches of the ATGC trees (see Discussion above and Figure 4), the GDE rates obtained here, even if strikingly high, should be considered as lower bound estimates.

### The supergenomes

The supergenome size estimates from the rates of repeated gene gain showed, in a broad agreement with previous observations, that some of the microbes possess well-defined closed supergenomes whereas other supergenomes appeared to be open. The typical closed supergenome size was estimated to be about tenfold larger than the characteristic genome size in the respective ATGC, indicating a large but clearly limited gene pool available to genome dynamics. The supergenome size does not show significant correlation with the overall gene dynamics, but seems to be associated with the phylogenetic depth of the ATGC tree and with the lifestyle, as open supergenomes are substantially more common among free-living compared to host-associated microbes. The accelerating sequencing of microbial genomes will put these estimates to test without much delay.

## Conclusions

The reconstruction of short-term GDEs shows that microbial genomes exist in a state of perennial flux, gaining, losing, expanding and contracting gene families. Typically, genome dynamics processes are rapid, with gains and losses of multiple gene families occurring within the time frame of a single nucleotide substitution per gene. Thus, gene flux is the dominant mode in microbial evolution such that microbes primarily differ from each other on the scale from static to highly dynamic. The rates of gene family gain and loss in most microbial groups are approximately an order of magnitude greater than the rates of expansion and contraction of pre-existing families, indicating that HGT is the principal source of new genes in prokaryote evolution. Overall, gene family loss notably prevails over gain, i.e. evolving genomes appear to spend more time contracting than expanding. It seems most likely that the gradual gene loss is compensated for by episodes of rapid gene gain; most such bursts are outside the evolutionary scale accessible through ATGCs although a few were detected. The absolute as well as relative rates of GDEs show remarkable variance among bacteria, spanning almost two orders of magnitude, and do not significantly depend on the ATGC-wide *dN*/*dS* estimates, the taxonomic affinity of microbes or their lifestyle. Conceivably, genome dynamics is highly sensitive to local ecological factors, the exact nature of which remains to be elucidated. The analysis of genome dynamics allowed us to estimate the size of microbial supergenomes, which in the majority of the analyzed microbial groups turned out to be large but closed, exceeding the characteristic genome size by about an order of magnitude, but for a minority of microbes appeared to be open.

## Methods

### The extended ATGC dataset

Genomic data was obtained from an updated version of the ATGC database [81] containing data from >4.5 million proteins present in >1,500 genomes of prokaryotes (approximately 60% of proteins and 62% of genomes from RefSeq as of June 2013) that met the same criteria as in the original ATGCs [58]. Specifically, these criteria include having at least 85% conserved synteny across any pair of genomes (alignable), and having synonymous substitution rate <1.5 (tight). Aside from the increase in the number and size of ATGCs due to the inclusion of new genomes of bacteria and archaea, the major difference between this and previous ATGC versions was the exclusion of lower-quality drafts of incomplete genomes. In addition, the pangenome of each ATGC is now represented by automatically derived COGs [67,68,82].

The COG construction was performed in two stages. First, COGs were constructed as clusters of bidirectional best-matching proteins, with the threshold *e*-value 1 × 10^−5^ and protein coverage of 75% [82]. Second, proteins unassigned in the first stage were added to the cluster that they match best using the COGNITOR method [83], with the stringent threshold *e*-value 1 × 10^−20^ and protein coverage of 75%.

The ATGCs also include pre-calculated *dN/dS* values [84] for all orthologous gene pairs from each pair of genomes. We analyzed 35 of the largest ATGCs (34 bacterial and one archaeal genomic cluster) that contained ten or more genomes (up to a maximum of 109; Additional file 2: Figures S1 and S15, Additional file 1: Table S1 and Additional file 3: Table S2). These selected ATGCs encompass many universal genes, i.e., genes that are present in all genomes within the ATGC.

### Species trees

First, a concatenated alignment of all universal genes with conserved synteny among species was constructed for each ATGC from the alignments of the respective protein sequences that were generated using MUSCLE [85] and converted back to the alignments of the respective nucleotide sequences using an in-house script. The concatenated alignments were used to reconstruct a species tree for each ATGC using the program FastTree [86] under the General Time Reversible (GTR) nucleotide substitution model [87]. The program Count that was employed for evolutionary reconstruction as described below [22], requires rooted phylogenetic trees as an input. Accordingly, all trees were rooted using the least-squares modification of the mid-point method [88].

### Phylogenetic birth-and-death analysis

The rates of gain, loss, expansion and reduction were estimated using the program Count [22]. This program requires two inputs, namely a matrix that contains the number of gene copies in each species and a rooted species tree, to calculate gain, loss, expansion and reduction rates. Count calculates these rates using a phylogenetic birth-and-death model that requires the following parameters: *κ* (rate of gene gain), *λ* (individual gene duplication rate) and *μ* (individual gene loss rate) (Additional file 2: Figure S16). Thus, a gene family of size *n* decreases at a rate *nμ* and increases at a rate (*κ* + *nλ*). The parameters (*κ,λ,μ*) are different for each gene family and across edges of the species tree. These parameters are computed by Count using ML optimization [75]. It is recommended that the parameters are optimized iteratively, in several rounds of increasing computational complexity, such that in each round the rates from the previous round are used as the starting point [22]. We optimized the parameters through 11 rounds of increasing complexity. The first two rounds started with uniform rates of gain and expansion and in the subsequent rounds the number of discrete categories for the gamma distribution (for gain, loss and expansion) increased from one to two for each type of event (Additional file 5: Table S4). The parameter values obtained in the final round were used to estimate the numbers of gains, losses, expansions and reductions for all gene families at different branches of the species tree. This final analysis was performed using the ‘posteriors’ option of Count, which analyzes and integrates several phylogenetic scenarios and calculates rates of gain, loss, expansion and reduction across all branches. The sum across all branches and across all families is taken as the estimate of the number of events across the entire history of a given group of organisms.

### Estimates of the supergenome size

Supergenome size was estimated using two different methods. We implemented the currently widely adopted binomial mixture method as well as the capture/recapture method [41]. We also utilized the posterior gain probabilities computed by Count to estimate the size of the reservoir (supergenome) from which gains originate. Only gains of non-ancestral families were included in the analysis. A family was designated as ancestral if the posterior probability of this family being present in the root node genome (the ancestor of the analyzed group) was above 0.5. The number of these ancestral families was added to the estimate of the supergenome size with the implicit assumption that they were gained before the common ancestor of the group came into existence.

The simplest procedure for estimating the supergenome size is to assume that every time a family is gained, it is drawn at random from a well-mixed reservoir of size *S* and maximize the probability

1 $$ L=\frac{C_S^P}{S^K} $$

that *P* distinct families are discovered as a result of *K* random independent samples from the reservoir, with respect to *S*. Here ${C}_S^P$ is a binomial coefficient. The total number of gains *K* is estimated as the sum of Count reported gain probabilities over all branches and families plus the sum of the probabilities of presence at the root node. Having an exact expression for the probability allows one to estimate the confidence region of the estimated supergenome size *S*. In reality acquisitions of genes from the supergenome are not random uncorrelated events. The effect of these correlations on the supergenome size estimates is likely to be complex and even its sign cannot be determined *a priori*. In the absence of ancillary information on gene gain correlations, the assumption of random independent gains provides a useful null model.

An alternative, more complex model of the reservoir posits that the probability *p*_*i*_ of gaining a family *i* can vary with *i*. The exact probability of observing *P* distinct families from *K* samples can no longer be computed explicitly. However, if we introduce the number *X*_*m*_ of families gained exactly *m* times, we can compute its expectation.

2 $$ {g}_m = {X}_m = {C}_K^m{\displaystyle \sum_{i=1}^S}{p}_i^m{\left(1-{p}_i\right)}^{K-m} $$

where ${C}_K^m$ is a binomial coefficient. Assuming that *X*_*m*_ is composed of a large number of independent binomials, it has a Poisson distribution. Therefore if the empirically observed number of families gained *m* times is *O*_*m*_, the log likelihood of observing *O*_1_, *O*_2_, etc. is

3 $$ \ln L \sim {\displaystyle \sum_{m=1}^K}\left(-{g}_m + {O}_m \ln {g}_m\right), $$

where *g*_*m*_ is computed via Equation 2. The gain probabilities *p*_*i*_ are parameterized using a power law distribution:

$$ {p}_i = \frac{A}{i^{\alpha }},\ i=1, \dots,\ S, $$

where *A* is a normalization constant that ensures that ${\displaystyle \sum_{i=1}^S}{p}_i = 1$. The power law above is the simplest non-trivial one-parameter distribution with a broad range of gain probabilities. The maximization of the approximate likelihood in Equation 3 with respect to *S* and *α* yields the estimate of the supergenome size *S* and the gain probability distribution in the supergenome characterized by *α*. The observed numbers *O*_*m*_ of multiple gains are computed by binning the total gain probabilities (including the ancestral branch gain probability), i.e. if the total Count reported gain probability for some family is between *m* − 0.5 and *m* + 0.5 for some integer *m*, *O*_*m*_ is incremented.

### Statistical analysis

Statistical analysis was performed in the R environment. Spearman rank correlations are reported. *P* values were obtained by a permutations test with 100,000 rounds of reshuffling. The input variables for the PCA were the total number of gains, losses, expansions and reductions per unit branch length in each ATGC (Table 1). These values were transformed into the logarithmic scale prior to the analysis. PCA was performed using the function princomp from the R statistical package. The sign of principal component 1 was inverted.

### Synteny distance

Within each ATGC, the pairwise synteny distance (*dY*) between genomes is defined as *1 – F*_*s*_ where *F*_*s*_ is the fraction of orthologs in syntenic genome segments [64]. Linear regression with a double logarithmic scale between pairwise synteny distance and nucleotide substitution distance was used to estimate the shuffling rate, which was reported for the nucleotide distance of 0.01 substitution/site.

### Bootstrap analysis of the genome dynamics event rates

The robustness of the median estimated GDE rates was assessed using bootstrap sampling of the estimated values of each type of GDE for the 35 ATGCs. Median values of 1,000 replicates of all distributions were collected and plotted in Additional file 2: Figure S3. The probability density of each distribution was calculated using the function density of the R statistical package.

## Additional files

Additional file 1: Table S1. List of ATGCs and genomes. Additional file 2: Figure S1. Scheme of the pipeline used in this study. We analyzed all ATGCs with ten or more species. The species tree was reconstructed with FastTree [87] from the concatenated alignment of universal COGs and rooted using the least-squares variation of the mid-point rooting [88]. The species tree and the phyletic distribution of COGs were used to calculate rates of genome dynamics with Count. **Figure S2.** Positive significant correlation of branch length (BL) with the number of **(a)** gains, **(b)** losses, **(c)** expansions and **(b)** reductions in individual tree branches. **Figure S3.** Distributions of the genome dynamics rates across the ATGCs and bootstrap analysis for **(a)** gains, **(b)** losses, **(c)** expansions and **(b)** reductions. **Figure S4.** Correlation of the rates of the four classes of GDE with the number of species in ATGCs. **Figure S5.** Relative genome dynamics by phylogenetic depth. **Figure S6.** Distributions of the genome dynamics rates across ATGCs. **(a)** Rates of gain, loss, expansion and reduction. **(b)** Rates of gain, loss, expansion and reduction considering only shallow branches (phylogenetic depth < 0.05). **(c)** Comparison of gain rates in all branches (solid green line) and in shallow branches only (dashed black line). **(d)** Comparison of loss rates in all branches (solid red line) and in shallow branches only (dashed black line). **(e)** Comparison of expansion rates in all branches (dashed green line) and in shallow branches only (dashed black line). **(f)** Comparison of reduction based in branches (dashed red line) and in shallow branches only (dashed black line). **Figure S7.** Correlation of overall genome dynamics with ATGC tree depth. **Figure S8.** Correlation of *dN*/*dS* and **(a)** gain, **(b)** loss, **(c)** expansion and **(d)** reduction rates in ATGCs. **Figure S9.** Correlation of GC content and **(a)** gain, **(b)** loss, **(c)** expansion and **(d)** reduction rates in ATGCs. **Figure S10.** Correlation of genome shuffling rate (*dY*) and **(a)** gain, **(b)** loss, **(c)** expansion and **(d)** reduction rates in ATGCs. **Figure S11.** Principal components analysis (PCA). **(a)** Values of the first and second components in the PCA across all ATGCs. **(b)** PCA loads. **(c)** Cumulative variance of the four components. **Figure S12.** Correlation of median number of protein coding genes (genome size) and **(a)** gain, **(b)** loss, **(c)** expansion and **(d)** reduction rates in ATGCs. **Figure S13.** Correlation of the estimated supergenome size (uniform model) and gene flux rates in ATGCs. **(a)** Supergenome size vs flux rate (all GDEs). **(b)** Supergenome size vs gain rate. **(c)** Supergenome size vs loss rate. **(d)** Supergenome size vs expansion rate. **(e)** Supergenome size vs reduction rate. **Figure S14.** Correlations between the estimated supergenome size (uniform model), *dN*/*dS* ratio, tree depth and average genome size in ATGCs. **(a)** Supergenome size vs tree depth. **(b)** Supergenome size vs *dN*/*dS*. **(c)** Tree depth vs *dN*/*dS*. **(d)** Genome size vs *dN*/*dS*. **Figure S15.** Frequency distribution of ATGCs by the number of species. **Figure S16.** Schematic representation of gains, losses, expansions and reductions. **(a)** Phylogenetic birth-and-death ML model used in Count. **(b)** Simple example to show that expansions and gains account for two possible evolutionary scenarios. Additional file 3: Table S2. Characteristics of ATGCs. Additional file 4: Table S3. Comparison of the present supergenome size estimates with previously published estimates. Additional file 5: Table S4. Parameters used in the Count analysis.

## Abbreviations

ATGC: alignable tight genome cluster
COG: cluster of orthologous genes
GDE: genome dynamics event
HGT: horizontal gene transfer
kb: kilobase
Mb: megabase
ML: maximum likelihood, PCA, principal component analysis
