# Supplementary material for: Genomes in turmoil: quantification of genome dynamics in prokaryote supergenomes
Source: BMC Biol. 2014 Aug 21;12:66. doi: 10.1186/s12915-014-0066-4 (PMC4166000; doi:10.1186/s12915-014-0066-4)
Supplement: Additional file 1: Table S1. — List of ATGCs and genomes. [file 12915_2014_66_MOESM1_ESM.docx]

**Table S1.** List of all genomes in the ATGCs

| ATGC001 | Citrobacter_koseri_ATCC_BAA_895_uid58143, Citrobacter_rodentium_ICC168_uid43089, Enterobacter_638_uid58727, Enterobacter_R4_368_uid208672, Enterobacter_asburiae_LF7a_uid72793, Enterobacter_cloacae_ATCC_13047_uid48363, Enterobacter_cloacae_ENHKU01_uid172463, Enterobacter_cloacae_EcWSU1_uid80739, Enterobacter_cloacae_SCF1_uid59969, Enterobacter_cloacae_dissolvens_SDM_uid168997, Escherichia_coli_042_uid161985, Escherichia_coli_536_uid58531, Escherichia_coli_55989_uid59383, Escherichia_coli_ABU_83972_uid161975, Escherichia_coli_APEC_O1_uid58623, Escherichia_coli_APEC_O78_uid187277, Escherichia_coli_ATCC_8739_uid58783, Escherichia_coli_BL21_DE3__uid161947, Escherichia_coli_BL21_DE3__uid161949, Escherichia_coli_BW2952_uid59391, Escherichia_coli_B_REL606_uid58803, Escherichia_coli_CFT073_uid57915, Escherichia_coli_DH1_uid161951, Escherichia_coli_DH1_uid162051, Escherichia_coli_E24377A_uid58395, Escherichia_coli_ED1a_uid59379, Escherichia_coli_ETEC_H10407_uid161993, Escherichia_coli_HS_uid58393, Escherichia_coli_IAI1_uid59377, Escherichia_coli_IAI39_uid59381, Escherichia_coli_IHE3034_uid162007, Escherichia_coli_KO11FL_uid162099, Escherichia_coli_KO11FL_uid52593, Escherichia_coli_K_12_substr__DH10B_uid58979, Escherichia_coli_K_12_substr__MDS42_uid193705, Escherichia_coli_K_12_substr__MG1655_uid57779, Escherichia_coli_K_12_substr__W3110_uid161931, Escherichia_coli_LF82_uid161965, Escherichia_coli_NA114_uid162139, Escherichia_coli_O103_H2_12009_uid41013, Escherichia_coli_O104_H4_2009EL_2050_uid175905, Escherichia_coli_O104_H4_2009EL_2071_uid176128, Escherichia_coli_O104_H4_2011C_3493_uid176127, Escherichia_coli_O111_H__11128_uid41023, Escherichia_coli_O127_H6_E2348_69_uid59343, Escherichia_coli_O157_H7_EC4115_uid59091, Escherichia_coli_O157_H7_EDL933_uid57831, Escherichia_coli_O157_H7_Sakai_uid57781, Escherichia_coli_O157_H7_TW14359_uid59235, Escherichia_coli_O26_H11_11368_uid41021, Escherichia_coli_O55_H7_CB9615_uid46655, Escherichia_coli_O55_H7_RM12579_uid162153, Escherichia_coli_O7_K1_CE10_uid162115, Escherichia_coli_O83_H1_NRG_857C_uid161987, Escherichia_coli_P12b_uid162061, Escherichia_coli_S88_uid62979, Escherichia_coli_SE11_uid59425, Escherichia_coli_SE15_uid161939, Escherichia_coli_SMS_3_5_uid58919, Escherichia_coli_UM146_uid162043, Escherichia_coli_UMN026_uid62981, Escherichia_coli_UMNK88_uid161991, Escherichia_coli_UTI89_uid58541, Escherichia_coli_W_uid162011, Escherichia_coli_W_uid162101, Escherichia_coli_Xuzhou21_uid163995, Escherichia_coli__BL21_Gold_DE3_pLysS_AG__uid59245, Escherichia_coli__clone_D_i14__uid162049, Escherichia_coli__clone_D_i2__uid162047, Escherichia_fergusonii_ATCC_35469_uid59375, Salmonella_bongori_NCTC_12419_uid70155, Salmonella_enterica_arizonae_serovar_62_z4_z23__uid58191, Salmonella_enterica_serovar_Agona_SL483_uid59431, Salmonella_enterica_serovar_Choleraesuis_SC_B67_uid58017, Salmonella_enterica_serovar_Dublin_CT_02021853_uid58917, Salmonella_enterica_serovar_Enteritidis_P125109_uid59247, Salmonella_enterica_serovar_Gallinarum_287_91_uid59249, Salmonella_enterica_serovar_Gallinarum_pullorum_RKS5078_uid87035, Salmonella_enterica_serovar_Heidelberg_B182_uid162195, Salmonella_enterica_serovar_Heidelberg_SL476_uid58973, Salmonella_enterica_serovar_Javiana_CFSAN001992_uid190101, Salmonella_enterica_serovar_Newport_SL254_uid58831, Salmonella_enterica_serovar_Paratyphi_A_AKU_12601_uid59269, Salmonella_enterica_serovar_Paratyphi_A_ATCC_9150_uid58201, Salmonella_enterica_serovar_Paratyphi_B_SPB7_uid59097, Salmonella_enterica_serovar_Paratyphi_C_RKS4594_uid59063, Salmonella_enterica_serovar_Schwarzengrund_CVM19633_uid58915, Salmonella_enterica_serovar_Typhi_CT18_uid57793, Salmonella_enterica_serovar_Typhi_P_stx_12_uid87001, Salmonella_enterica_serovar_Typhi_Ty21a_uid201427, Salmonella_enterica_serovar_Typhi_Ty2_uid57973, Salmonella_enterica_serovar_Typhimurium_14028S_uid86059, Salmonella_enterica_serovar_Typhimurium_798_uid158047, Salmonella_enterica_serovar_Typhimurium_LT2_uid57799, Salmonella_enterica_serovar_Typhimurium_SL1344_uid86645, Salmonella_enterica_serovar_Typhimurium_ST4_74_uid84393, Salmonella_enterica_serovar_Typhimurium_T000240_uid84397, Salmonella_enterica_serovar_Typhimurium_U288_uid198746, Salmonella_enterica_serovar_Typhimurium_UK_1_uid87049, Salmonella_enterica_serovar_Typhimurium_uid86061, Shigella_boydii_CDC_3083_94_uid58415, Shigella_boydii_Sb227_uid58215, Shigella_dysenteriae_Sd197_uid58213, Shigella_flexneri_2002017_uid159233, Shigella_flexneri_2a_2457T_uid57991, Shigella_flexneri_2a_301_uid62907, Shigella_flexneri_5_8401_uid58583, Shigella_sonnei_53G_uid84383, Shigella_sonnei_Ss046_uid58217 |
| --- | --- |
| ATGC002 | Enterobacter_aerogenes_EA1509E_uid187411, Enterobacter_aerogenes_KCTC_2190_uid68103, Klebsiella_oxytoca_E718_uid170256, Klebsiella_oxytoca_KCTC_1686_uid83159, Klebsiella_pneumoniae_1084_uid174151, Klebsiella_pneumoniae_342_uid59145, Klebsiella_pneumoniae_HS11286_uid84387, Klebsiella_pneumoniae_KCTC_2242_uid162147, Klebsiella_pneumoniae_MGH_78578_uid57619, Klebsiella_pneumoniae_NTUH_K2044_uid59073, Klebsiella_variicola_At_22_uid42113 |
| ATGC003 | Streptococcus_mitis_B6_uid46097, Streptococcus_pneumoniae_670_6B_uid52533, Streptococcus_pneumoniae_70585_uid59125, Streptococcus_pneumoniae_AP200_uid52453, Streptococcus_pneumoniae_ATCC_700669_uid59287, Streptococcus_pneumoniae_CGSP14_uid59181, Streptococcus_pneumoniae_D39_uid58581, Streptococcus_pneumoniae_G54_uid59167, Streptococcus_pneumoniae_Hungary19A_6_uid59117, Streptococcus_pneumoniae_INV104_uid162039, Streptococcus_pneumoniae_INV200_uid162035, Streptococcus_pneumoniae_JJA_uid59121, Streptococcus_pneumoniae_OXC141_uid162037, Streptococcus_pneumoniae_P1031_uid59123, Streptococcus_pneumoniae_R6_uid57859, Streptococcus_pneumoniae_SPNA45_uid174986, Streptococcus_pneumoniae_ST556_uid162191, Streptococcus_pneumoniae_TCH8431_19A_uid49735, Streptococcus_pneumoniae_TIGR4_uid57857, Streptococcus_pneumoniae_Taiwan19F_14_uid59119, Streptococcus_pneumoniae_gamPNI0373_uid175861, Streptococcus_pseudopneumoniae_IS7493_uid71153 |
| ATGC004 | Streptococcus_dysgalactiae_equisimilis_AC_2713_uid178644, Streptococcus_dysgalactiae_equisimilis_ATCC_12394_uid161979, Streptococcus_dysgalactiae_equisimilis_GGS_124_uid59103, Streptococcus_dysgalactiae_equisimilis_RE378_uid176684, Streptococcus_pyogenes_A20_uid178106, Streptococcus_pyogenes_Alab49_uid162171, Streptococcus_pyogenes_M1_476_uid193766, Streptococcus_pyogenes_M1_GAS_uid57845, Streptococcus_pyogenes_MGAS10270_uid58571, Streptococcus_pyogenes_MGAS10394_uid58105, Streptococcus_pyogenes_MGAS10750_uid58575, Streptococcus_pyogenes_MGAS15252_uid158037, Streptococcus_pyogenes_MGAS1882_uid158061, Streptococcus_pyogenes_MGAS2096_uid58573, Streptococcus_pyogenes_MGAS315_uid57911, Streptococcus_pyogenes_MGAS5005_uid58337, Streptococcus_pyogenes_MGAS6180_uid58335, Streptococcus_pyogenes_MGAS8232_uid57871, Streptococcus_pyogenes_MGAS9429_uid58569, Streptococcus_pyogenes_Manfredo_uid57847, Streptococcus_pyogenes_NZ131_uid59035, Streptococcus_pyogenes_SSI_1_uid57895 |
| ATGC005 | Streptococcus_suis_05ZYH33_uid58663, Streptococcus_suis_98HAH33_uid58665, Streptococcus_suis_A7_uid162111, Streptococcus_suis_BM407_uid59321, Streptococcus_suis_D12_uid162127, Streptococcus_suis_D9_uid162125, Streptococcus_suis_GZ1_uid161937, Streptococcus_suis_JS14_uid162095, Streptococcus_suis_P1_7_uid32235, Streptococcus_suis_S735_uid174333, Streptococcus_suis_SC070731_uid193769, Streptococcus_suis_SC84_uid59323, Streptococcus_suis_SS12_uid162123, Streptococcus_suis_ST1_uid167482, Streptococcus_suis_ST3_uid66327, Streptococcus_suis_TL13_uid203123 |
| ATGC014 | Bacillus_anthracis_A0248_uid59385, Bacillus_anthracis_Ames_uid57909, Bacillus_anthracis_CDC_684_uid59303, Bacillus_anthracis_H9401_uid162021, Bacillus_anthracis_Sterne_uid58091, Bacillus_anthracis__Ames_Ancestor__uid58083, Bacillus_cereus_03BB102_uid59299, Bacillus_cereus_AH187_uid58753, Bacillus_cereus_AH820_uid58751, Bacillus_cereus_ATCC_10987_uid57673, Bacillus_cereus_ATCC_14579_uid57975, Bacillus_cereus_B4264_uid58757, Bacillus_cereus_E33L_uid58103, Bacillus_cereus_F837_76_uid83611, Bacillus_cereus_FRI_35_uid173403, Bacillus_cereus_G9842_uid58759, Bacillus_cereus_NC7401_uid82815, Bacillus_cereus_Q1_uid58529, Bacillus_cereus_biovar_anthracis_CI_uid50615, Bacillus_thuringiensis_Al_Hakam_uid58795, Bacillus_thuringiensis_BMB171_uid49135, Bacillus_thuringiensis_Bt407_uid177931, Bacillus_thuringiensis_HD_771_uid173374, Bacillus_thuringiensis_HD_789_uid173860, Bacillus_thuringiensis_MC28_uid176369, Bacillus_thuringiensis_serovar_IS5056_uid190186, Bacillus_thuringiensis_serovar_chinensis_CT_43_uid158151, Bacillus_thuringiensis_serovar_finitimus_YBT_020_uid158875, Bacillus_thuringiensis_serovar_konkukian_97_27_uid58089, Bacillus_thuringiensis_serovar_kurstaki_HD73_uid189188, Bacillus_weihenstephanensis_KBAB4_uid58315 |
| ATGC015 | Bacillus_JS_uid162189, Bacillus_amyloliquefaciens_DSM_7_uid53535, Bacillus_amyloliquefaciens_FZB42_uid58271, Bacillus_amyloliquefaciens_IT_45_uid181617, Bacillus_amyloliquefaciens_LL3_uid158133, Bacillus_amyloliquefaciens_TA208_uid158701, Bacillus_amyloliquefaciens_XH7_uid158881, Bacillus_amyloliquefaciens_Y2_uid165195, Bacillus_amyloliquefaciens_plantarum_AS43_3_uid183682, Bacillus_amyloliquefaciens_plantarum_CAU_B946_uid84215, Bacillus_amyloliquefaciens_plantarum_UCMB5036_uid190705, Bacillus_amyloliquefaciens_plantarum_YAU_B9601_Y2_uid159001, Bacillus_atrophaeus_1942_uid59887, Bacillus_subtilis_168_uid57675, Bacillus_subtilis_6051_HGW_uid193706, Bacillus_subtilis_BAB_1_uid195461, Bacillus_subtilis_BSP1_uid184010, Bacillus_subtilis_BSn5_uid62463, Bacillus_subtilis_QB928_uid173926, Bacillus_subtilis_RO_NN_1_uid158879, Bacillus_subtilis_XF_1_uid189187, Bacillus_subtilis_natto_BEST195_uid183001, Bacillus_subtilis_spizizenii_TU_B_10_uid73967, Bacillus_subtilis_spizizenii_W23_uid51879 |
| ATGC021 | Chlamydia_trachomatis_434_Bu_uid61633, Chlamydia_trachomatis_A2497_uid159863, Chlamydia_trachomatis_A2497_uid159993, Chlamydia_trachomatis_A_363_uid196769, Chlamydia_trachomatis_A_5291_uid196770, Chlamydia_trachomatis_A_HAR_13_uid58333, Chlamydia_trachomatis_B_Jali20_OT_uid59351, Chlamydia_trachomatis_B_TZ1A828_OT_uid59349, Chlamydia_trachomatis_D_EC_uid159881, Chlamydia_trachomatis_D_LC_uid159879, Chlamydia_trachomatis_D_SotonD5_uid196773, Chlamydia_trachomatis_D_UW_3_CX_uid57637, Chlamydia_trachomatis_E_11023_uid161369, Chlamydia_trachomatis_E_150_uid161403, Chlamydia_trachomatis_E_Bour_uid196775, Chlamydia_trachomatis_E_SW3_uid167483, Chlamydia_trachomatis_F_SW4_uid167484, Chlamydia_trachomatis_F_SW5_uid167485, Chlamydia_trachomatis_G_11074_uid161409, Chlamydia_trachomatis_G_11222_uid161361, Chlamydia_trachomatis_G_9301_uid161377, Chlamydia_trachomatis_G_9768_uid161353, Chlamydia_trachomatis_G_SotonG1_uid196779, Chlamydia_trachomatis_IU824_uid193712, Chlamydia_trachomatis_IU888_uid193713, Chlamydia_trachomatis_Ia_SotonIa1_uid196780, Chlamydia_trachomatis_K_SotonK1_uid196782, Chlamydia_trachomatis_L1_115_uid196784, Chlamydia_trachomatis_L1_224_uid196785, Chlamydia_trachomatis_L1_440_LN_uid196783, Chlamydia_trachomatis_L2_25667R_uid196786, Chlamydia_trachomatis_L2_434_Bu_f__uid198644, Chlamydia_trachomatis_L2_434_Bu_i__uid198643, Chlamydia_trachomatis_L2b_795_uid196791, Chlamydia_trachomatis_L2b_8200_07_uid196787, Chlamydia_trachomatis_L2b_Ams1_uid196792, Chlamydia_trachomatis_L2b_UCH_1_proctitis_uid61635, Chlamydia_trachomatis_L2b_UCH_2_uid196788, Chlamydia_trachomatis_L2c_uid68843, Chlamydia_trachomatis_L3_404_LN_uid196797, Chlamydia_trachomatis_Sweden2_uid161995, Chlamydia_trachomatis_uid196771, Chlamydia_trachomatis_uid196776, Chlamydia_trachomatis_uid196781 |
| ATGC022 | Chlamydia_psittaci_01DC12_uid179070, Chlamydia_psittaci_84_55_uid175571, Chlamydia_psittaci_GR9_uid175572, Chlamydia_psittaci_M56_uid175576, Chlamydia_psittaci_MN_uid175573, Chlamydia_psittaci_VS225_uid175574, Chlamydia_psittaci_WC_uid175577, Chlamydia_psittaci_WS_RT_E30_uid175575, Chlamydophila_abortus_S26_3_uid57963, Chlamydophila_psittaci_01DC11_uid159527, Chlamydophila_psittaci_02DC15_uid159521, Chlamydophila_psittaci_08DC60_uid159525, Chlamydophila_psittaci_6BC_uid159845, Chlamydophila_psittaci_6BC_uid63621, Chlamydophila_psittaci_C19_98_uid159523, Chlamydophila_psittaci_CP3_uid175578, Chlamydophila_psittaci_Mat116_uid189026, Chlamydophila_psittaci_NJ1_uid175579, Chlamydophila_psittaci_RD1_uid162063 |
| ATGC025 | Mycobacterium_africanum_GM041182_uid68839, Mycobacterium_bovis_AF2122_97_uid57695, Mycobacterium_bovis_BCG_Korea_1168P_uid189029, Mycobacterium_bovis_BCG_Mexico_uid86889, Mycobacterium_bovis_BCG_Pasteur_1173P2_uid58781, Mycobacterium_bovis_BCG_Tokyo_172_uid59281, Mycobacterium_canettii_CIPT_140010059_uid70731, Mycobacterium_canettii_CIPT_140060008_uid184829, Mycobacterium_canettii_CIPT_140070008_uid184832, Mycobacterium_canettii_CIPT_140070010_uid184828, Mycobacterium_canettii_CIPT_140070017_uid184830, Mycobacterium_tuberculosis_Beijing_NITR203_uid197218, Mycobacterium_tuberculosis_CAS_NITR204_uid202217, Mycobacterium_tuberculosis_CCDC5079_uid161943, Mycobacterium_tuberculosis_CCDC5079_uid203790, Mycobacterium_tuberculosis_CCDC5180_uid161941, Mycobacterium_tuberculosis_CDC1551_uid57775, Mycobacterium_tuberculosis_CTRI_2_uid161997, Mycobacterium_tuberculosis_EAI5_NITR206_uid202218, Mycobacterium_tuberculosis_Erdman___ATCC_35801_uid193763, Mycobacterium_tuberculosis_F11_uid58417, Mycobacterium_tuberculosis_H37Ra_uid58853, Mycobacterium_tuberculosis_H37Rv_uid170532, Mycobacterium_tuberculosis_H37Rv_uid57777, Mycobacterium_tuberculosis_Haarlem3_NITR202_uid202216, Mycobacterium_tuberculosis_KZN_1435_uid59069, Mycobacterium_tuberculosis_KZN_4207_uid83619, Mycobacterium_tuberculosis_KZN_605_uid54947, Mycobacterium_tuberculosis_RGTB327_uid157907, Mycobacterium_tuberculosis_RGTB423_uid162179, Mycobacterium_tuberculosis_UT205_uid162183, Mycobacterium_tuberculosis_uid185758 |
| ATGC033 | Mycoplasma_gallisepticum_CA06_2006_052_5_2P_uid172630, Mycoplasma_gallisepticum_F_uid162001, Mycoplasma_gallisepticum_NC06_2006_080_5_2P_uid172629, Mycoplasma_gallisepticum_NC08_2008_031_4_3P_uid172631, Mycoplasma_gallisepticum_NC95_13295_2_2P_uid172625, Mycoplasma_gallisepticum_NC96_1596_4_2P_uid172626, Mycoplasma_gallisepticum_NY01_2001_047_5_1P_uid172627, Mycoplasma_gallisepticum_R_high__uid161999, Mycoplasma_gallisepticum_R_low__uid57993, Mycoplasma_gallisepticum_VA94_7994_1_7P_uid172624, Mycoplasma_gallisepticum_WI01_2001_043_13_2P_uid172628 |
| ATGC046 | Candidatus_Rickettsia_amblyommii_GAT_30V_uid156845, Rickettsia_africae_ESF_5_uid58799, Rickettsia_akari_Hartford_uid58161, Rickettsia_australis_Cutlack_uid158039, Rickettsia_canadensis_CA410_uid88063, Rickettsia_canadensis_McKiel_uid58159, Rickettsia_conorii_Malish_7_uid57633, Rickettsia_felis_URRWXCal2_uid58331, Rickettsia_heilongjiangensis_054_uid70839, Rickettsia_japonica_YH_uid73963, Rickettsia_massiliae_AZT80_uid86751, Rickettsia_massiliae_MTU5_uid58801, Rickettsia_montanensis_OSU_85_930_uid158043, Rickettsia_parkeri_Portsmouth_uid158045, Rickettsia_peacockii_Rustic_uid59301, Rickettsia_philipii_364D_uid89383, Rickettsia_prowazekii_Breinl_uid196851, Rickettsia_prowazekii_BuV67_CWPP_uid158063, Rickettsia_prowazekii_Chernikova_uid158053, Rickettsia_prowazekii_Dachau_uid158057, Rickettsia_prowazekii_GvV257_uid158051, Rickettsia_prowazekii_Katsinyian_uid158055, Rickettsia_prowazekii_Madrid_E_uid61565, Rickettsia_prowazekii_NMRC_Madrid_E_uid196850, Rickettsia_prowazekii_Rp22_uid161945, Rickettsia_prowazekii_RpGvF24_uid158065, Rickettsia_rhipicephali_3_7_female6_CWPP_uid156977, Rickettsia_rickettsii_Arizona_uid86655, Rickettsia_rickettsii_Brazil_uid88069, Rickettsia_rickettsii_Colombia_uid86653, Rickettsia_rickettsii_Hauke_uid86659, Rickettsia_rickettsii_Hino_uid86657, Rickettsia_rickettsii_Hlp_2_uid88067, Rickettsia_rickettsii_Iowa_uid58961, Rickettsia_rickettsii__Sheila_Smith__uid58027, Rickettsia_slovaca_13_B_uid82369, Rickettsia_slovaca_D_CWPP_uid158159, Rickettsia_typhi_B9991CWPP_uid158357, Rickettsia_typhi_TH1527_uid158161, Rickettsia_typhi_Wilmington_uid58063 |
| ATGC052 | Helicobacter_acinonychis_Sheeba_uid58685, Helicobacter_cetorum_MIT_99_5656_uid162215, Helicobacter_pylori_2017_uid161151, Helicobacter_pylori_2018_uid161159, Helicobacter_pylori_26695_uid178201, Helicobacter_pylori_26695_uid57787, Helicobacter_pylori_35A_uid49903, Helicobacter_pylori_51_uid161925, Helicobacter_pylori_83_uid161153, Helicobacter_pylori_908_uid159985, Helicobacter_pylori_Aklavik117_uid182201, Helicobacter_pylori_Aklavik86_uid182202, Helicobacter_pylori_B38_uid59415, Helicobacter_pylori_B8_uid49873, Helicobacter_pylori_Cuz20_uid159987, Helicobacter_pylori_ELS37_uid158157, Helicobacter_pylori_F16_uid161145, Helicobacter_pylori_F30_uid159991, Helicobacter_pylori_F32_uid161139, Helicobacter_pylori_F57_uid161143, Helicobacter_pylori_G27_uid59305, Helicobacter_pylori_Gambia94_24_uid159493, Helicobacter_pylori_HPAG1_uid58517, Helicobacter_pylori_HUP_B14_uid162213, Helicobacter_pylori_India7_uid161149, Helicobacter_pylori_J99_uid57789, Helicobacter_pylori_Lithuania75_uid159491, Helicobacter_pylori_OK113_uid193715, Helicobacter_pylori_OK310_uid193716, Helicobacter_pylori_P12_uid59327, Helicobacter_pylori_PeCan18_uid162211, Helicobacter_pylori_PeCan4_uid53539, Helicobacter_pylori_Puno120_uid159611, Helicobacter_pylori_Puno135_uid161157, Helicobacter_pylori_Rif1_uid178202, Helicobacter_pylori_Rif2_uid178203, Helicobacter_pylori_SJM180_uid53541, Helicobacter_pylori_SNT49_uid159615, Helicobacter_pylori_Sat464_uid159467, Helicobacter_pylori_Shi112_uid162207, Helicobacter_pylori_Shi169_uid162209, Helicobacter_pylori_Shi417_uid162205, Helicobacter_pylori_Shi470_uid59165, Helicobacter_pylori_SouthAfrica7_uid159989, Helicobacter_pylori_UM032_uid203025, Helicobacter_pylori_UM037_uid203027, Helicobacter_pylori_UM066_uid203028, Helicobacter_pylori_UM299_uid203026, Helicobacter_pylori_XZ274_uid165869, Helicobacter_pylori_uid159983, Helicobacter_pylori_v225d_uid159639 |
| ATGC054 | Staphylococcus_aureus_04_02981_uid161969, Staphylococcus_aureus_08BA02176_uid175257, Staphylococcus_aureus_11819_97_uid159981, Staphylococcus_aureus_71193_uid162141, Staphylococcus_aureus_CC45_uid209174, Staphylococcus_aureus_COL_uid57797, Staphylococcus_aureus_ECT_R_2_uid159389, Staphylococcus_aureus_ED133_uid159689, Staphylococcus_aureus_ED98_uid41455, Staphylococcus_aureus_HO_5096_0412_uid162163, Staphylococcus_aureus_JH1_uid58457, Staphylococcus_aureus_JH9_uid58455, Staphylococcus_aureus_JKD6008_uid159855, Staphylococcus_aureus_JKD6159_uid159691, Staphylococcus_aureus_LGA251_uid159391, Staphylococcus_aureus_M013_uid88065, Staphylococcus_aureus_M1_uid197263, Staphylococcus_aureus_MRSA252_uid57839, Staphylococcus_aureus_MSSA476_uid57841, Staphylococcus_aureus_MW2_uid57903, Staphylococcus_aureus_Mu3_uid58817, Staphylococcus_aureus_Mu50_uid57835, Staphylococcus_aureus_N315_uid57837, Staphylococcus_aureus_NCTC_8325_uid57795, Staphylococcus_aureus_Newman_uid58839, Staphylococcus_aureus_RF122_uid57661, Staphylococcus_aureus_ST228_10388_uid193754, Staphylococcus_aureus_ST228_10497_uid193755, Staphylococcus_aureus_ST228_15532_uid193756, Staphylococcus_aureus_ST228_16035_uid193757, Staphylococcus_aureus_ST228_18412_uid193760, Staphylococcus_aureus_ST398_uid159247, Staphylococcus_aureus_T0131_uid159861, Staphylococcus_aureus_TCH60_uid159859, Staphylococcus_aureus_TW20_uid159241, Staphylococcus_aureus_USA300_FPR3757_uid58555, Staphylococcus_aureus_USA300_TCH1516_uid58925, Staphylococcus_aureus_VC40_uid88071, Staphylococcus_aureus_uid193758, Staphylococcus_aureus_uid193759, Staphylococcus_aureus_uid193761 |
| ATGC056 | Lactobacillus_casei_ATCC_334_uid57985, Lactobacillus_casei_BD_II_uid162119, Lactobacillus_casei_BL23_uid59237, Lactobacillus_casei_LC2W_uid162121, Lactobacillus_casei_W56_uid178736, Lactobacillus_casei_Zhang_uid50673, Lactobacillus_rhamnosus_ATCC_8530_uid162169, Lactobacillus_rhamnosus_GG_uid161983, Lactobacillus_rhamnosus_GG_uid59313, Lactobacillus_rhamnosus_Lc_705_uid59315 |
| ATGC067 | Corynebacterium_pseudotuberculosis_1002_uid159677, Corynebacterium_pseudotuberculosis_1_06_A_uid159665, Corynebacterium_pseudotuberculosis_258_uid167260, Corynebacterium_pseudotuberculosis_267_uid162175, Corynebacterium_pseudotuberculosis_316_uid89381, Corynebacterium_pseudotuberculosis_31_uid162167, Corynebacterium_pseudotuberculosis_3_99_5_uid83609, Corynebacterium_pseudotuberculosis_42_02_A_uid159669, Corynebacterium_pseudotuberculosis_C231_uid159675, Corynebacterium_pseudotuberculosis_CIP_52_97_uid159667, Corynebacterium_pseudotuberculosis_Cp162_uid168258, Corynebacterium_pseudotuberculosis_FRC41_uid50585, Corynebacterium_pseudotuberculosis_I19_uid159673, Corynebacterium_pseudotuberculosis_P54B96_uid157909, Corynebacterium_pseudotuberculosis_PAT10_uid159671 |
| ATGC068 | Corynebacterium_diphtheriae_241_uid83607, Corynebacterium_diphtheriae_31A_uid84309, Corynebacterium_diphtheriae_BH8_uid84311, Corynebacterium_diphtheriae_CDCE_8392_uid84295, Corynebacterium_diphtheriae_HC01_uid84297, Corynebacterium_diphtheriae_HC02_uid84317, Corynebacterium_diphtheriae_HC03_uid84299, Corynebacterium_diphtheriae_HC04_uid84301, Corynebacterium_diphtheriae_INCA_402_uid83605, Corynebacterium_diphtheriae_NCTC_13129_uid57691, Corynebacterium_diphtheriae_PW8_uid84303, Corynebacterium_diphtheriae_VA01_uid84305 |
| ATGC072 | Pseudomonas_ND6_uid167583, Pseudomonas_entomophila_L48_uid58639, Pseudomonas_putida_BIRD_1_uid162055, Pseudomonas_putida_DOT_T1E_uid171260, Pseudomonas_putida_F1_uid58355, Pseudomonas_putida_GB_1_uid58735, Pseudomonas_putida_H8234_uid208673, Pseudomonas_putida_HB3267_uid184078, Pseudomonas_putida_KT2440_uid57843, Pseudomonas_putida_NBRC_14164_uid208670, Pseudomonas_putida_S16_uid68747, Pseudomonas_putida_W619_uid58651 |
| ATGC082 | Clostridium_botulinum_A2_Kyoto_uid59229, Clostridium_botulinum_A3_Loch_Maree_uid59149, Clostridium_botulinum_A_ATCC_19397_uid58927, Clostridium_botulinum_A_ATCC_3502_uid61579, Clostridium_botulinum_A_Hall_uid58931, Clostridium_botulinum_B1_Okra_uid59147, Clostridium_botulinum_Ba4_657_uid59173, Clostridium_botulinum_F_230613_uid159513, Clostridium_botulinum_F_Langeland_uid58929, Clostridium_botulinum_H04402_065_uid162091 |
| ATGC089 | Burkholderia_mallei_ATCC_23344_uid57725, Burkholderia_mallei_NCTC_10229_uid58383, Burkholderia_mallei_NCTC_10247_uid58385, Burkholderia_mallei_SAVP1_uid58387, Burkholderia_pseudomallei_1026b_uid162511, Burkholderia_pseudomallei_1106a_uid58515, Burkholderia_pseudomallei_1710b_uid58391, Burkholderia_pseudomallei_668_uid58389, Burkholderia_pseudomallei_BPC006_uid174460, Burkholderia_pseudomallei_K96243_uid57733, Burkholderia_pseudomallei_MSHR346_uid55259, Burkholderia_thailandensis_E264_uid58081, Burkholderia_thailandensis_MSMB121_uid201037 |
| ATGC090 | Burkholderia_383_uid58073, Burkholderia_KJ006_uid165871, Burkholderia_ambifaria_AMMD_uid58303, Burkholderia_ambifaria_MC40_6_uid58701, Burkholderia_cenocepacia_AU_1054_uid58371, Burkholderia_cenocepacia_HI2424_uid58369, Burkholderia_cenocepacia_J2315_uid57953, Burkholderia_cenocepacia_MC0_3_uid58769, Burkholderia_cepacia_GG4_uid173858, Burkholderia_multivorans_ATCC_17616_uid58697, Burkholderia_multivorans_ATCC_17616_uid58909, Burkholderia_vietnamiensis_G4_uid58075 |
| ATGC094 | Sulfolobus_islandicus_HVE10_4_uid162067, Sulfolobus_islandicus_LAL14_1_uid197216, Sulfolobus_islandicus_L_D_8_5_uid43679, Sulfolobus_islandicus_L_S_2_15_uid58871, Sulfolobus_islandicus_M_14_25_uid58849, Sulfolobus_islandicus_M_16_27_uid58851, Sulfolobus_islandicus_M_16_4_uid58841, Sulfolobus_islandicus_REY15A_uid162071, Sulfolobus_islandicus_Y_G_57_14_uid58923, Sulfolobus_islandicus_Y_N_15_51_uid58825, Sulfolobus_solfataricus_98_2_uid167998, Sulfolobus_solfataricus_P2_uid57721 |
| ATGC105 | Bifidobacterium_breve_ACS_071_V_Sch8b_uid158863, Bifidobacterium_breve_UCC2003_uid193702, Bifidobacterium_longum_BBMN68_uid60163, Bifidobacterium_longum_DJO10A_uid58833, Bifidobacterium_longum_JCM_1217_uid62695, Bifidobacterium_longum_JDM301_uid49131, Bifidobacterium_longum_KACC_91563_uid158861, Bifidobacterium_longum_NCC2705_uid57939, Bifidobacterium_longum_infantis_157F_uid62693, Bifidobacterium_longum_infantis_ATCC_15697_uid159865, Bifidobacterium_longum_infantis_ATCC_15697_uid58677 |
| ATGC106 | Bifidobacterium_animalis_ATCC_25527_uid162513, Bifidobacterium_animalis_lactis_AD011_uid58911, Bifidobacterium_animalis_lactis_B420_uid163691, Bifidobacterium_animalis_lactis_BB_12_uid158871, Bifidobacterium_animalis_lactis_BLC1_uid158867, Bifidobacterium_animalis_lactis_Bi_07_uid163693, Bifidobacterium_animalis_lactis_Bl_04_uid59359, Bifidobacterium_animalis_lactis_CNCM_I_2494_uid158869, Bifidobacterium_animalis_lactis_DSM_10140_uid59357, Bifidobacterium_animalis_lactis_V9_uid158865 |
| ATGC109 | Listeria_innocua_Clip11262_uid61567, Listeria_ivanovii_PAM_55_uid73473, Listeria_monocytogenes_07PF0776_uid162185, Listeria_monocytogenes_08_5923_uid43727, Listeria_monocytogenes_10403S_uid54461, Listeria_monocytogenes_ATCC_19117_uid175109, Listeria_monocytogenes_Clip80459_uid59317, Listeria_monocytogenes_EGD_e_uid61583, Listeria_monocytogenes_FSL_R2_561_uid54441, Listeria_monocytogenes_Finland_1998_uid54443, Listeria_monocytogenes_HCC23_uid59203, Listeria_monocytogenes_J0161_uid54459, Listeria_monocytogenes_L312_uid175768, Listeria_monocytogenes_La111_uid193768, Listeria_monocytogenes_M7_uid162131, Listeria_monocytogenes_N53_1_uid193767, Listeria_monocytogenes_SLCC2372_uid174872, Listeria_monocytogenes_SLCC2376_uid175111, Listeria_monocytogenes_SLCC2378_uid175105, Listeria_monocytogenes_SLCC2479_uid175108, Listeria_monocytogenes_SLCC2540_uid175106, Listeria_monocytogenes_SLCC5850_uid175110, Listeria_monocytogenes_SLCC7179_uid175107, Listeria_monocytogenes_serotype_1_2b_SLCC2755_uid52455, Listeria_monocytogenes_serotype_4a_L99_uid161953, Listeria_monocytogenes_serotype_4b_F2365_uid57689, Listeria_monocytogenes_serotype_4b_LL195_uid182103, Listeria_monocytogenes_serotype_7_SLCC2482_uid174871, Listeria_monocytogenes_uid43671, Listeria_seeligeri_serovar_1_2b_SLCC3954_uid46215, Listeria_welshimeri_serovar_6b_SLCC5334_uid61605 |
| ATGC121 | Shewanella_ANA_3_uid58347, Shewanella_MR_4_uid58345, Shewanella_MR_7_uid58343, Shewanella_W3_18_1_uid58341, Shewanella_baltica_BA175_uid52601, Shewanella_baltica_OS117_uid162025, Shewanella_baltica_OS155_uid58259, Shewanella_baltica_OS185_uid58743, Shewanella_baltica_OS195_uid58261, Shewanella_baltica_OS223_uid58775, Shewanella_baltica_OS678_uid50553, Shewanella_oneidensis_MR_1_uid57949, Shewanella_putrefaciens_200_uid161927, Shewanella_putrefaciens_CN_32_uid58267 |
| ATGC128 | Yersinia_enterocolitica_8081_uid57741, Yersinia_enterocolitica_palearctica_105_5R_r__uid63663, Yersinia_enterocolitica_palearctica_Y11_uid162069, Yersinia_pestis_A1122_uid158119, Yersinia_pestis_Angola_uid58485, Yersinia_pestis_Antiqua_uid58607, Yersinia_pestis_CO92_uid57621, Yersinia_pestis_D106004_uid158071, Yersinia_pestis_D182038_uid158073, Yersinia_pestis_KIM_10_uid57875, Yersinia_pestis_Nepal516_uid58609, Yersinia_pestis_Pestoides_F_uid58619, Yersinia_pestis_Z176003_uid47317, Yersinia_pestis_biovar_Medievalis_Harbin_35_uid158537, Yersinia_pestis_biovar_Microtus_91001_uid58037, Yersinia_pseudotuberculosis_IP_31758_uid58487, Yersinia_pseudotuberculosis_IP_32953_uid58157, Yersinia_pseudotuberculosis_PB1__uid59153, Yersinia_pseudotuberculosis_YPIII_uid59151 |
| ATGC135 | Xanthomonas_axonopodis_Xac29_1_uid193774, Xanthomonas_axonopodis_citri_306_uid57889, Xanthomonas_axonopodis_citrumelo_F1_uid73179, Xanthomonas_campestris_8004_uid57595, Xanthomonas_campestris_ATCC_33913_uid57887, Xanthomonas_campestris_raphani_756C_uid159539, Xanthomonas_campestris_uid61643, Xanthomonas_campestris_vesicatoria_85_10_uid58321, Xanthomonas_citri_Aw12879_uid194444, Xanthomonas_oryzae_KACC_10331_uid58155, Xanthomonas_oryzae_MAFF_311018_uid58547, Xanthomonas_oryzae_PXO99A_uid59131, Xanthomonas_oryzae_oryzicola_BLS256_uid54411 |
| ATGC137 | Brucella_abortus_A13334_uid83615, Brucella_abortus_S19_uid58873, Brucella_abortus_bv__1_9_941_uid58019, Brucella_canis_ATCC_23365_uid59009, Brucella_canis_HSK_A52141_uid83613, Brucella_melitensis_ATCC_23457_uid59241, Brucella_melitensis_M28_uid158857, Brucella_melitensis_M5_90_uid158855, Brucella_melitensis_NI_uid158853, Brucella_melitensis_biovar_Abortus_2308_uid62937, Brucella_melitensis_bv__1_16M_uid57735, Brucella_microti_CCM_4915_uid59319, Brucella_ovis_ATCC_25840_uid58113, Brucella_pinnipedialis_B2_94_uid71131, Brucella_suis_1330_uid159871, Brucella_suis_1330_uid57927, Brucella_suis_ATCC_23445_uid59015, Brucella_suis_VBI22_uid83617, Ochrobactrum_anthropi_ATCC_49188_uid58921 |
| ATGC138 | Neisseria_gonorrhoeae_FA_1090_uid57611, Neisseria_gonorrhoeae_NCCP11945_uid59191, Neisseria_gonorrhoeae_TCDC_NG08107_uid161097, Neisseria_lactamica_020_06_uid60851, Neisseria_meningitidis_053442_uid58587, Neisseria_meningitidis_8013_uid161967, Neisseria_meningitidis_FAM18_uid57825, Neisseria_meningitidis_G2136_uid162085, Neisseria_meningitidis_H44_76_uid162083, Neisseria_meningitidis_M01_240149_uid162079, Neisseria_meningitidis_M01_240355_uid162075, Neisseria_meningitidis_M04_240196_uid162081, Neisseria_meningitidis_MC58_uid57817, Neisseria_meningitidis_NZ_05_33_uid162077, Neisseria_meningitidis_WUE_2594_uid162093, Neisseria_meningitidis_Z2491_uid57819, Neisseria_meningitidis_alpha14_uid61649, Neisseria_meningitidis_alpha710_uid161971 |
| ATGC139 | Francisella_TX077308_uid68321, Francisella_cf__novicida_3523_uid162107, Francisella_cf__novicida_Fx1_uid162105, Francisella_noatunensis_orientalis_Toba_04_uid164779, Francisella_novicida_U112_uid58499, Francisella_philomiragia_ATCC_25017_uid59105, Francisella_tularensis_FSC198_uid58693, Francisella_tularensis_NE061598_uid161973, Francisella_tularensis_SCHU_S4_uid57589, Francisella_tularensis_TI0902_uid89373, Francisella_tularensis_TIGB03_uid89379, Francisella_tularensis_WY96_3418_uid58811, Francisella_tularensis_holarctica_F92_uid181998, Francisella_tularensis_holarctica_FSC200_uid54341, Francisella_tularensis_holarctica_FTNF002_00_uid58999, Francisella_tularensis_holarctica_LVS_uid58595, Francisella_tularensis_holarctica_OSU18_uid58687, Francisella_tularensis_mediasiatica_FSC147_uid58939 |
| ATGC144 | Campylobacter_jejuni_81116_uid58771, Campylobacter_jejuni_81_176_uid58503, Campylobacter_jejuni_IA3902_uid159531, Campylobacter_jejuni_ICDCCJ07001_uid61249, Campylobacter_jejuni_M1_uid159535, Campylobacter_jejuni_NCTC_11168_BN148_uid174152, Campylobacter_jejuni_NCTC_11168___ATCC_700819_uid57587, Campylobacter_jejuni_PT14_uid176499, Campylobacter_jejuni_RM1221_uid57899, Campylobacter_jejuni_S3_uid159533, Campylobacter_jejuni_doylei_269_97_uid58671 |
| ATGC153 | Acinetobacter_baumannii_1656_2_uid158677, Acinetobacter_baumannii_AB0057_uid59083, Acinetobacter_baumannii_AB307_0294_uid59271, Acinetobacter_baumannii_ACICU_uid58765, Acinetobacter_baumannii_ATCC_17978_uid58731, Acinetobacter_baumannii_AYE_uid61637, Acinetobacter_baumannii_D1279779_uid190222, Acinetobacter_baumannii_MDR_TJ_uid162739, Acinetobacter_baumannii_MDR_ZJ06_uid158685, Acinetobacter_baumannii_SDF_uid61601, Acinetobacter_baumannii_TCDC_AB0715_uid158679, Acinetobacter_baumannii_TYTH_1_uid176498, Acinetobacter_calcoaceticus_PHEA_2_uid83123, Acinetobacter_oleivorans_DR1_uid50119 |
| ATGC163 | Propionibacterium_acnes_266_uid162059, Propionibacterium_acnes_6609_uid162137, Propionibacterium_acnes_ATCC_11828_uid162177, Propionibacterium_acnes_C1_uid176501, Propionibacterium_acnes_HL096PA1_uid198524, Propionibacterium_acnes_KPA171202_uid58101, Propionibacterium_acnes_SK137_uid48071, Propionibacterium_acnes_TypeIA2_P_acn17_uid80735, Propionibacterium_acnes_TypeIA2_P_acn31_uid80733, Propionibacterium_acnes_TypeIA2_P_acn33_uid80745, Propionibacterium_avidum_44067_uid197361 |
| ATGC186 | Legionella_pneumophila_2300_99_Alcoy_uid48801, Legionella_pneumophila_ATCC_43290_uid86885, Legionella_pneumophila_Corby_uid58733, Legionella_pneumophila_HL06041035_uid170534, Legionella_pneumophila_Lens_uid58209, Legionella_pneumophila_Lorraine_uid170535, Legionella_pneumophila_Paris_uid58211, Legionella_pneumophila_Philadelphia_1_uid193710, Legionella_pneumophila_Philadelphia_1_uid57609, Legionella_pneumophila_Thunder_Bay_uid206517 |
