# Supplementary material for: Genomes in turmoil: quantification of genome dynamics in prokaryote supergenomes
Source: BMC Biol. 2014 Aug 21;12:66. doi: 10.1186/s12915-014-0066-4 (PMC4166000; doi:10.1186/s12915-014-0066-4)
Supplement: Additional file 2: Figure S1. — Scheme of the pipeline used in this study. We analyzed all ATGCs with ten or more species. The species tree was reconstructed with FastTree [87] from the concatenated alignment of universal COGs and rooted using the least-squares variation of the mid-point rooting [88]. The species tree and the phyletic distribution of COGs were used to calculate rates of genome dynamics with Count. Figure S2. Positive significant correlation of branch length (BL) with the number of (a) gains, (b) losses, (c) expansions and (b) reductions in individual tree branches. Figure S3. Distributions of the genome dynamics rates across the ATGCs and bootstrap analysis for (a) gains, (b) losses, (c) expansions and (b) reductions. Figure S4. Correlation of the rates of the four classes of GDE with the number of species in ATGCs. Figure S5. Relative genome dynamics by phylogenetic depth. Figure S6. Distributions of the genome dynamics rates across ATGCs. (a) Rates of gain, loss, expansion and reduction. (b) Rates of gain, loss, expansion and reduction considering only shallow branches (phylogenetic depth < 0.05). (c) Comparison of gain rates in all branches (solid green line) and in shallow branches only (dashed black line). (d) Comparison of loss rates in all branches (solid red line) and in shallow branches only (dashed black line). (e) Comparison of expansion rates in all branches (dashed green line) and in shallow branches only (dashed black line). (f) Comparison of reduction based in branches (dashed red line) and in shallow branches only (dashed black line). Figure S7. Correlation of overall genome dynamics with ATGC tree depth. Figure S8. Correlation of dN/dS and (a) gain, (b) loss, (c) expansion and (d) reduction rates in ATGCs. Figure S9. Correlation of GC content and (a) gain, (b) loss, (c) expansion and (d) reduction rates in ATGCs. Figure S10. Correlation of genome shuffling rate (dY) and (a) gain, (b) loss, (c) expansion and (d) reduction rates in ATGCs. Figure S11. Principal compo [file 12915_2014_66_MOESM2_ESM.pptx]

## Slide 1
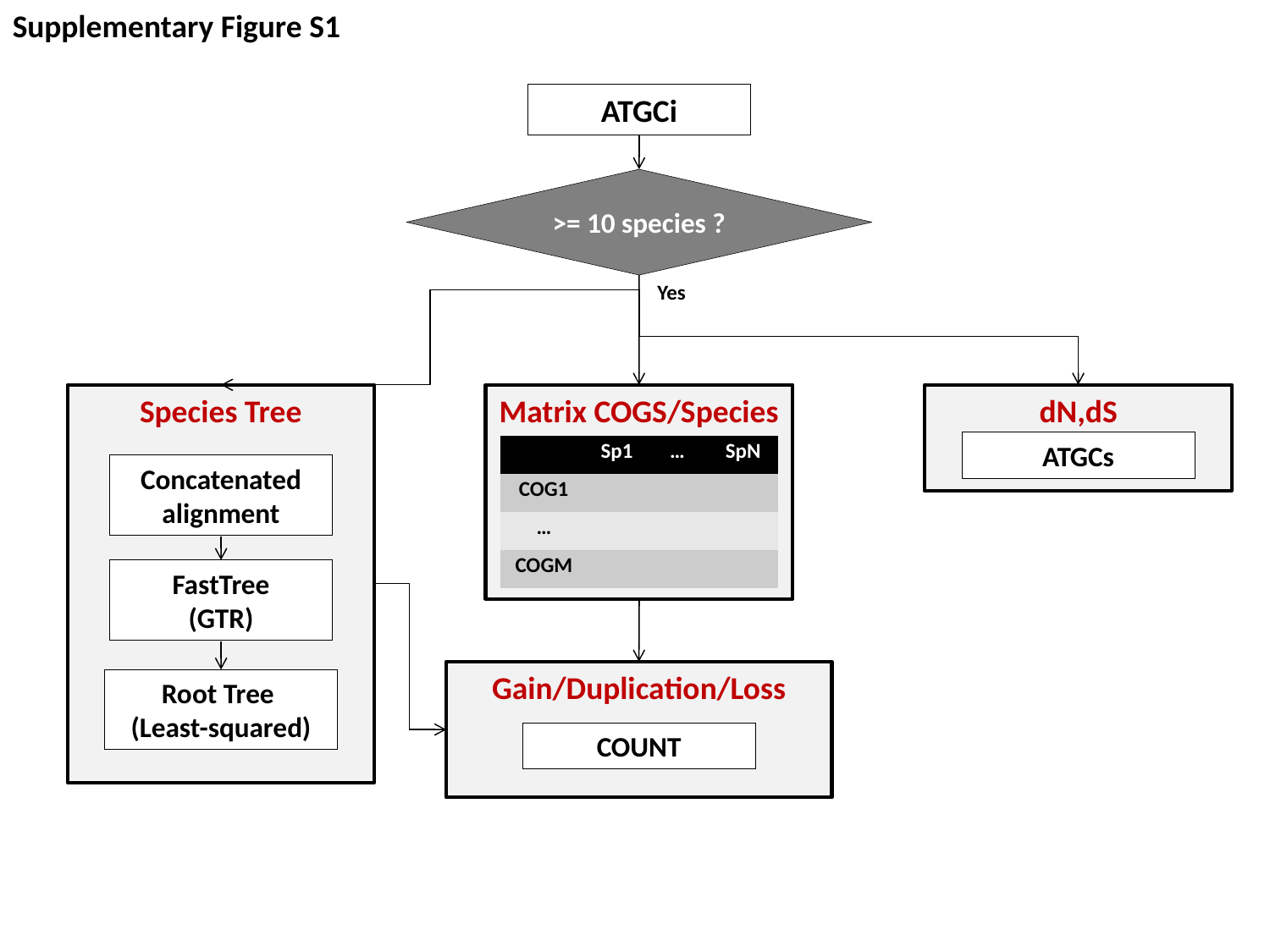

Supplementary Figure S1
ATGCi
>= 10 species ?
Yes
Species Tree
Concatenated alignment
FastTree
(GTR)
Root Tree
(Least-squared)
Matrix COGS/Species
dN,dS
ATGCs
| | Sp1 | … | SpN |
| --- | --- | --- | --- |
| COG1 | | | |
| … | | | |
| COGM | | | |
Gain/Duplication/Loss
COUNT

## Slide 2
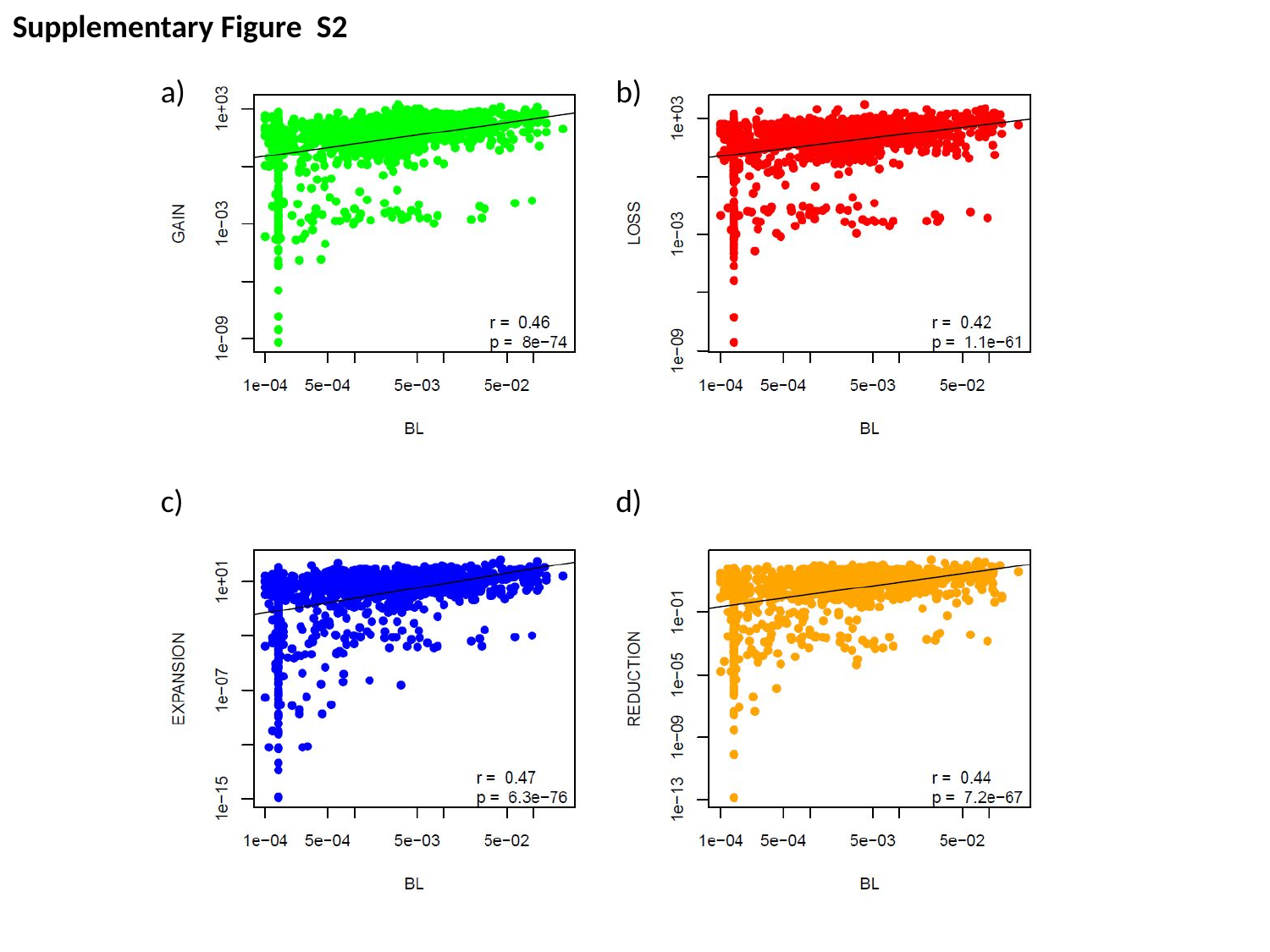

Supplementary Figure S2
a)
b)
c)
d)

## Slide 3
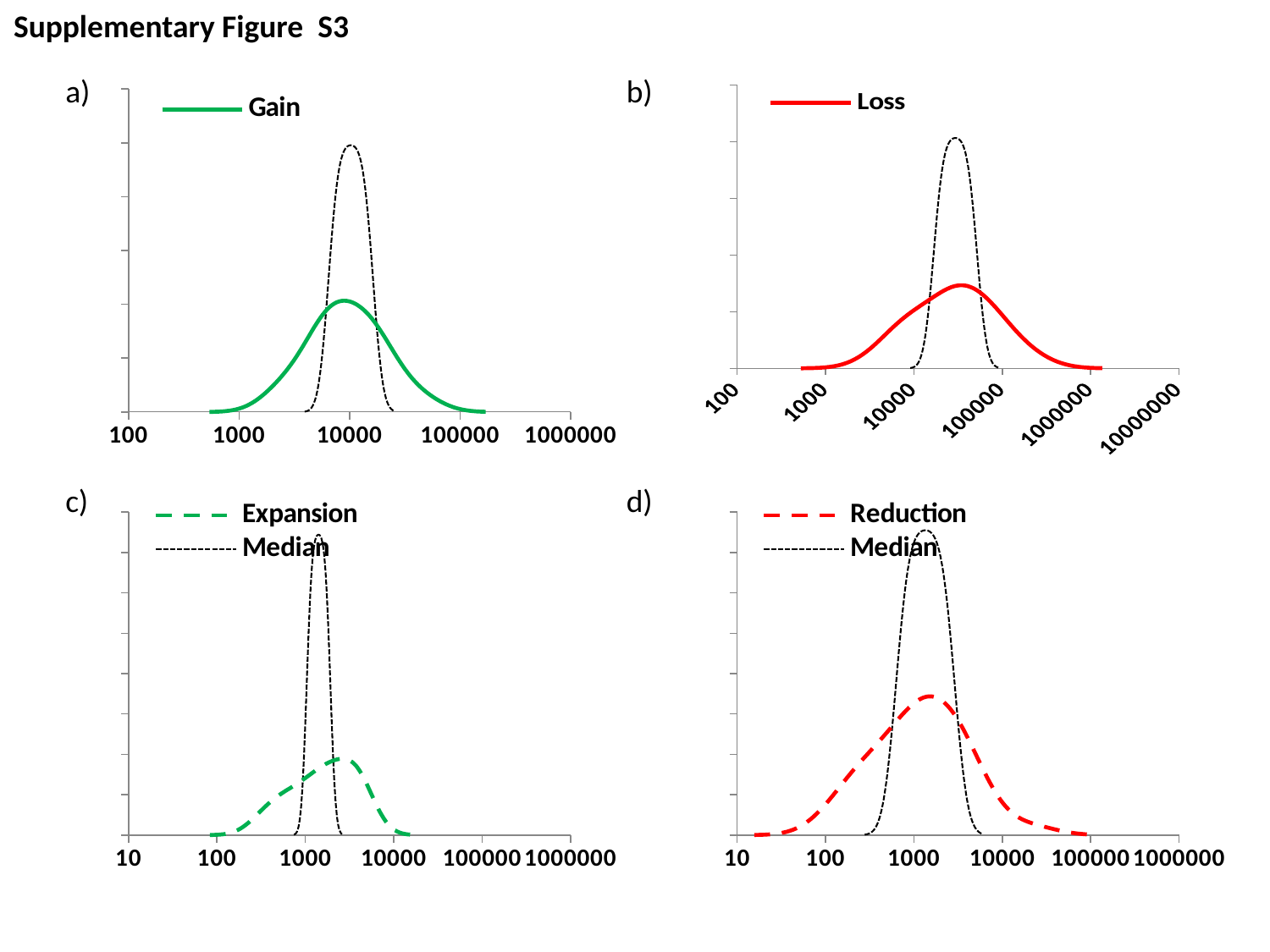

Supplementary Figure S3
### Chart
| Category | Gain | Median |
|---|---|---|
### Chart
| Category | Loss | Median |
|---|---|---|a)
b)
c)
d)
### Chart
| Category | Expansion | Median |
|---|---|---|
### Chart
| Category | Reduction | Median |
|---|---|---|

## Slide 4
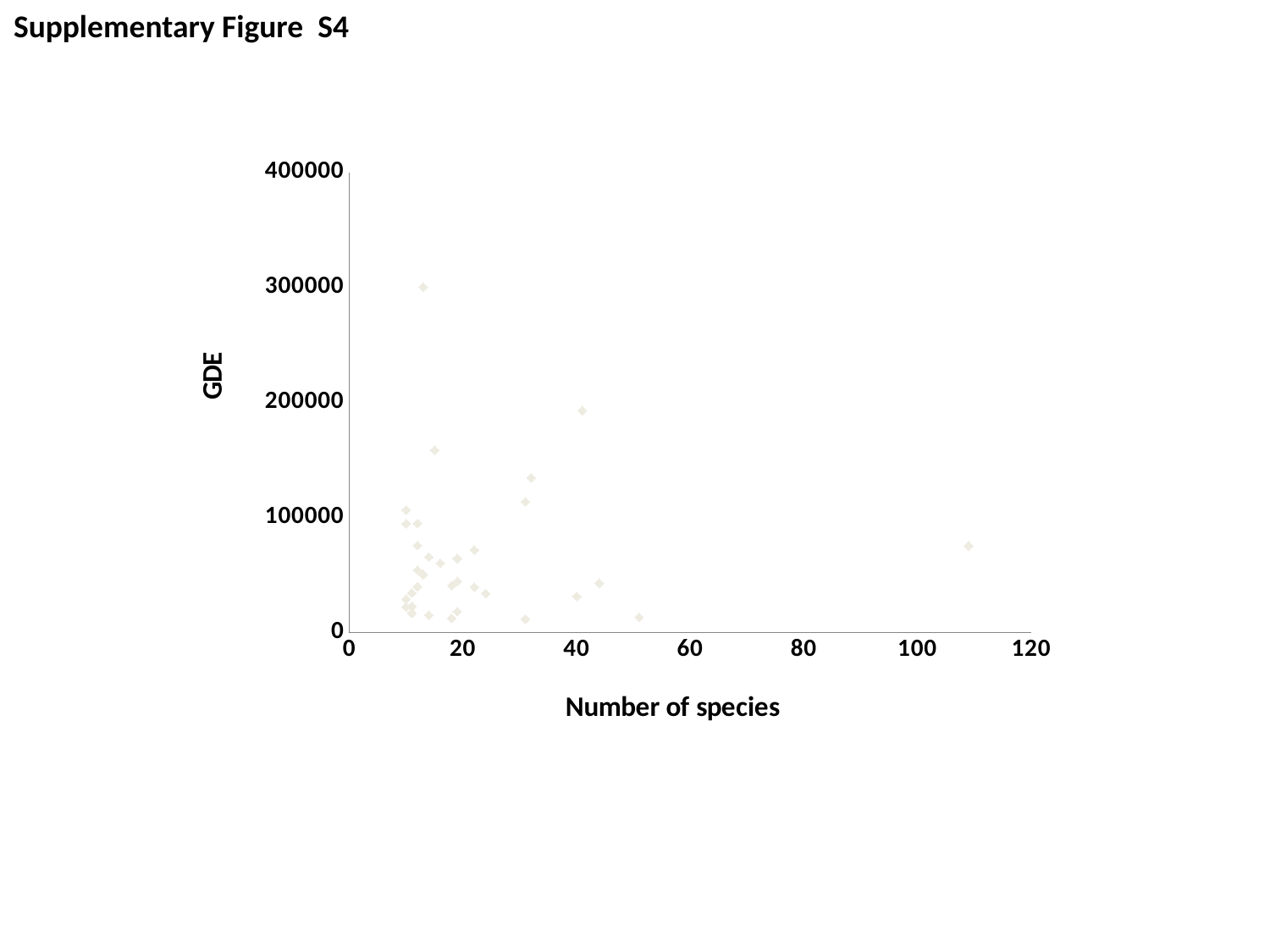

Supplementary Figure S4
### Chart
| Category | |
|---|---|

## Slide 5
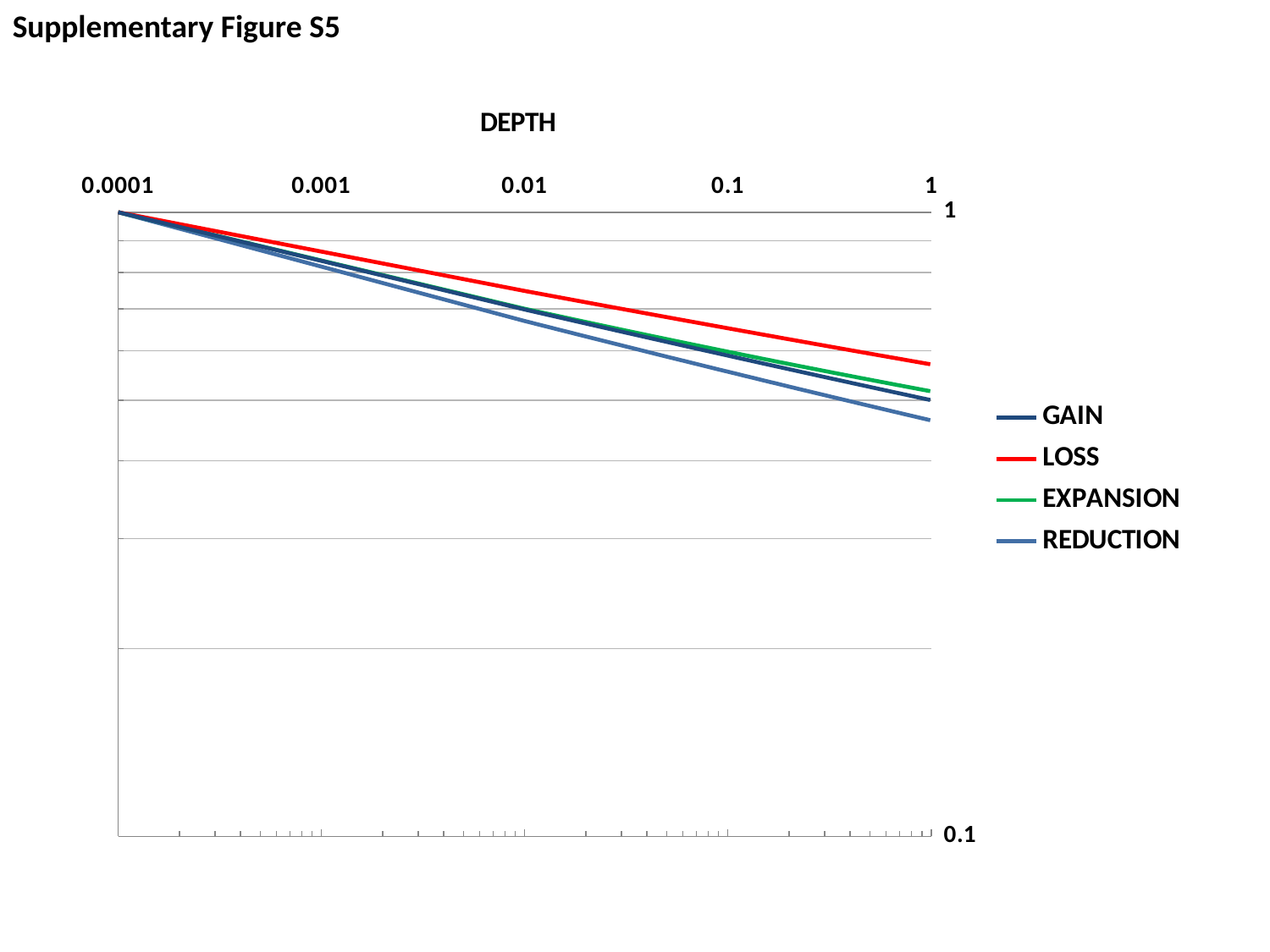

Supplementary Figure S5
### Chart
| Category | GAIN | LOSS | EXPANSION | REDUCTION |
|---|---|---|---|---|

## Slide 6
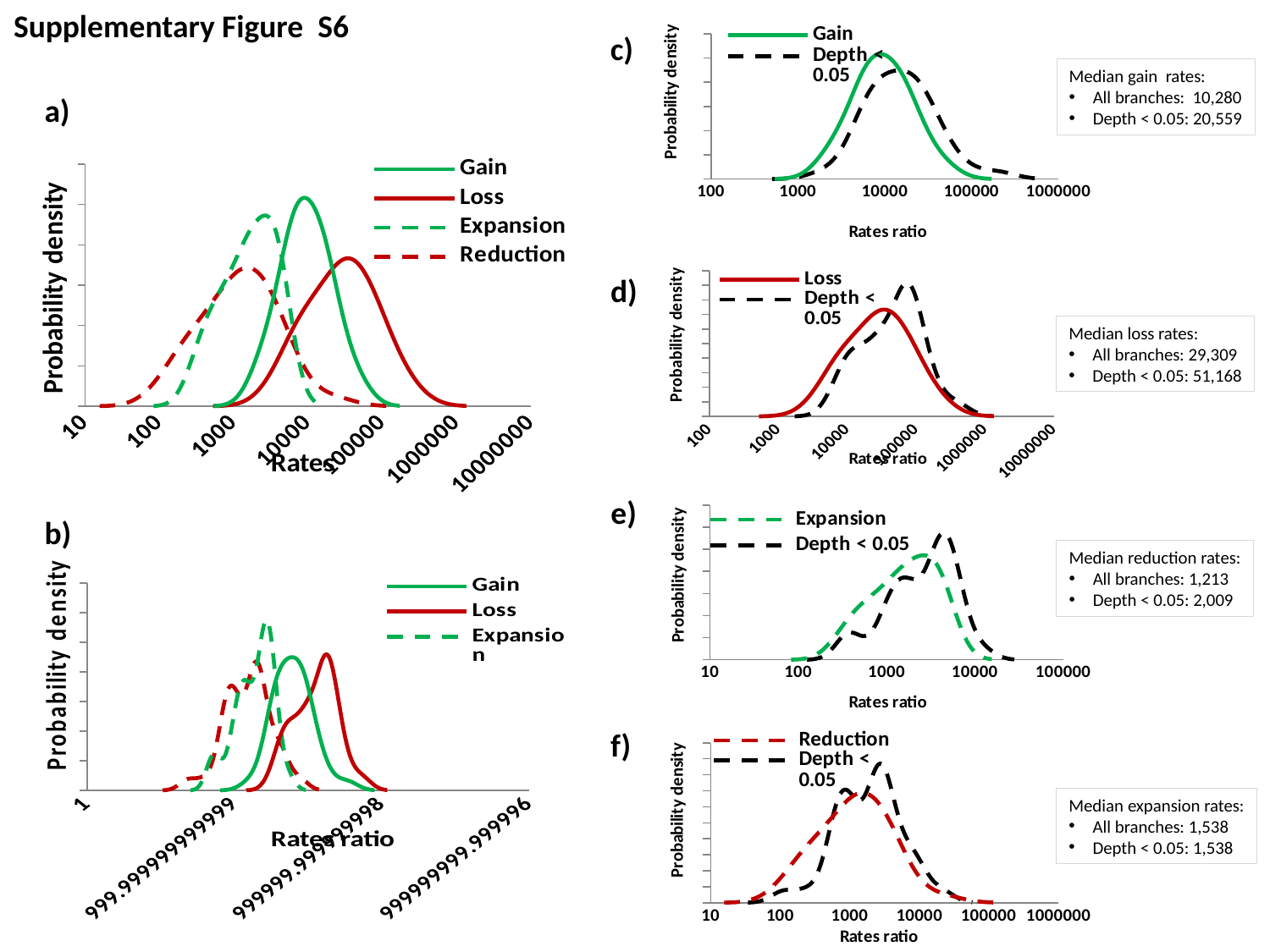

Supplementary Figure S6
c)
### Chart
| Category | Depth < 0.05 | Gain |
|---|---|---|Median gain rates:
All branches: 10,280
Depth < 0.05: 20,559
a)
### Chart
| Category | Reduction | Expansion | Gain | Loss |
|---|---|---|---|---|
### Chart
| Category | Depth < 0.05 | Loss |
|---|---|---|d)
Median loss rates:
All branches: 29,309
Depth < 0.05: 51,168
e)
### Chart
| Category | Depth < 0.05 | Expansion |
|---|---|---|b)
Median reduction rates:
All branches: 1,213
Depth < 0.05: 2,009
### Chart
| Category | Reduction | Expansion | Gain | Loss |
|---|---|---|---|---|f)
### Chart
| Category | Depth < 0.05 | Reduction |
|---|---|---|Median expansion rates:
All branches: 1,538
Depth < 0.05: 1,538

## Slide 7
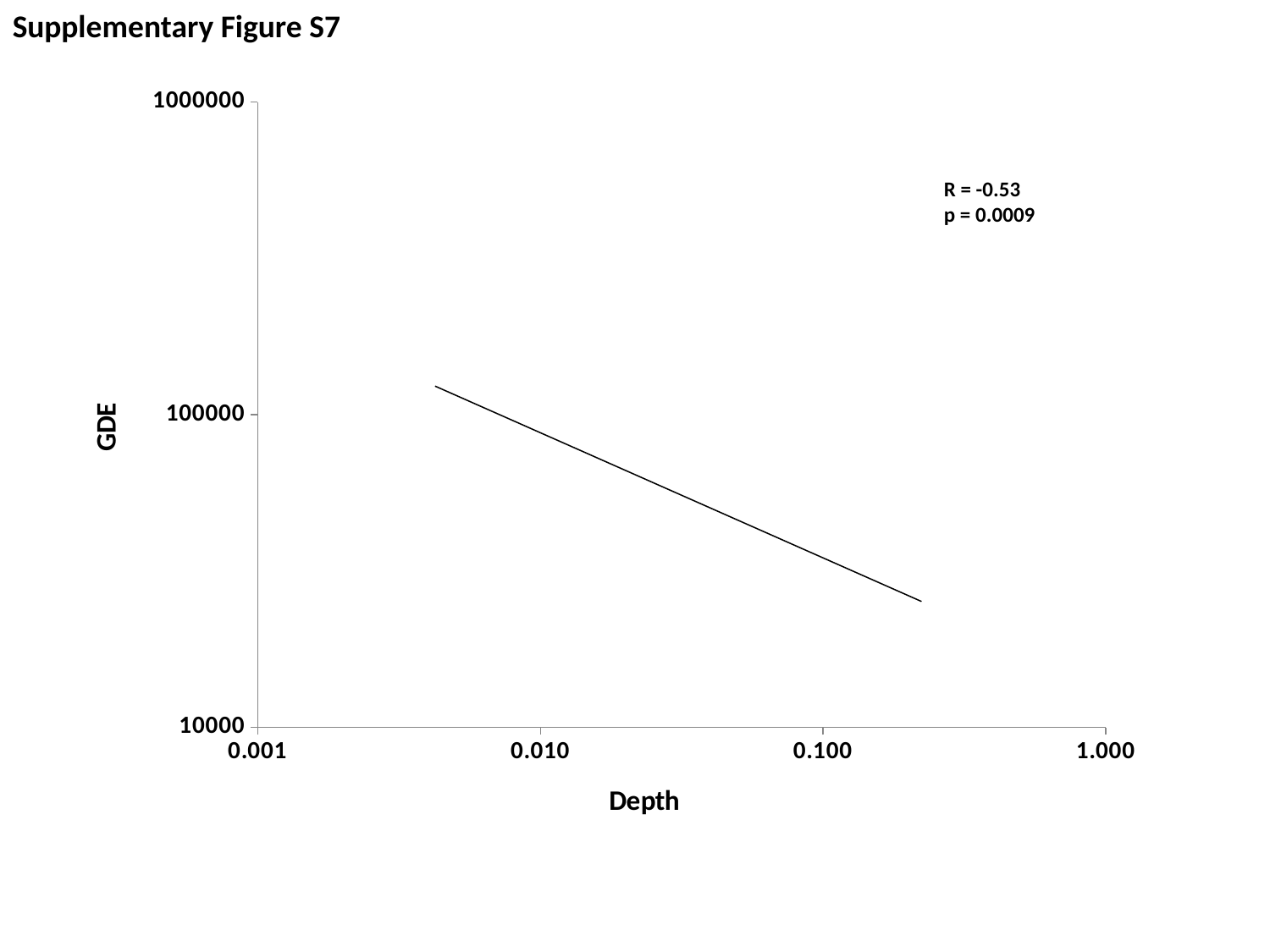

Supplementary Figure S7
### Chart
| Category | |
|---|---|R = -0.53
p = 0.0009

## Slide 8
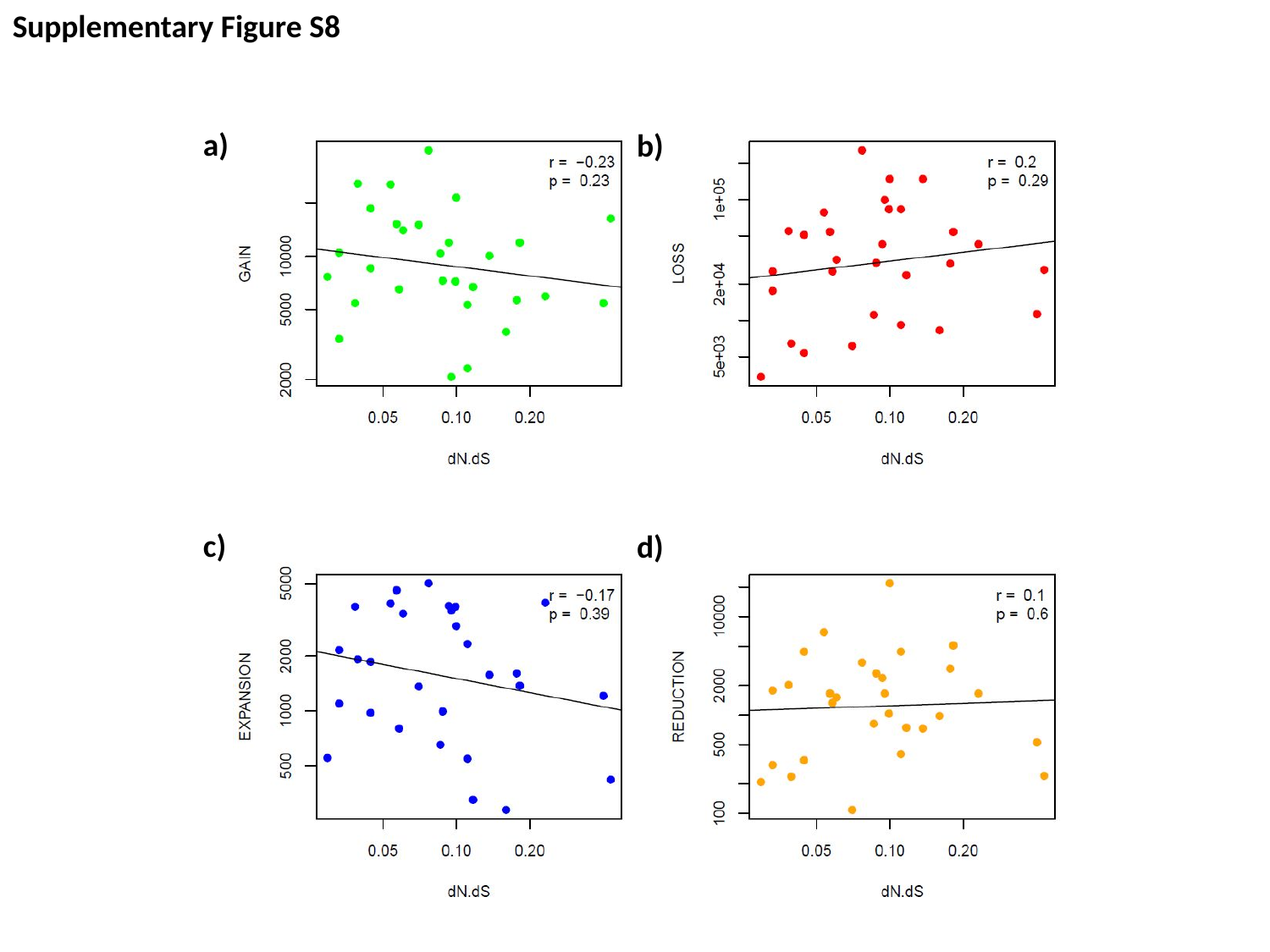

Supplementary Figure S8
a)
b)
c)
d)

## Slide 9
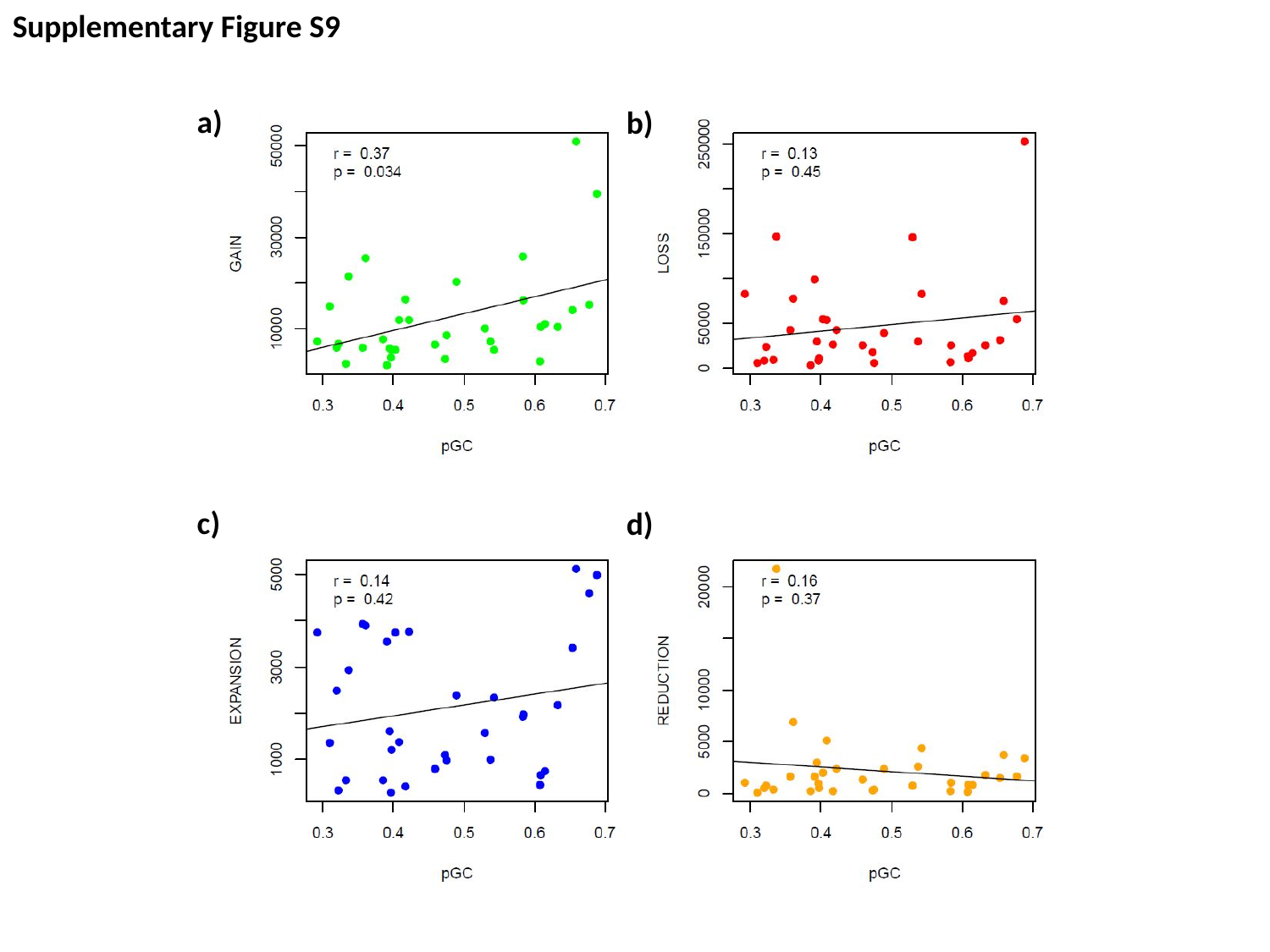

Supplementary Figure S9
a)
b)
c)
d)

## Slide 10
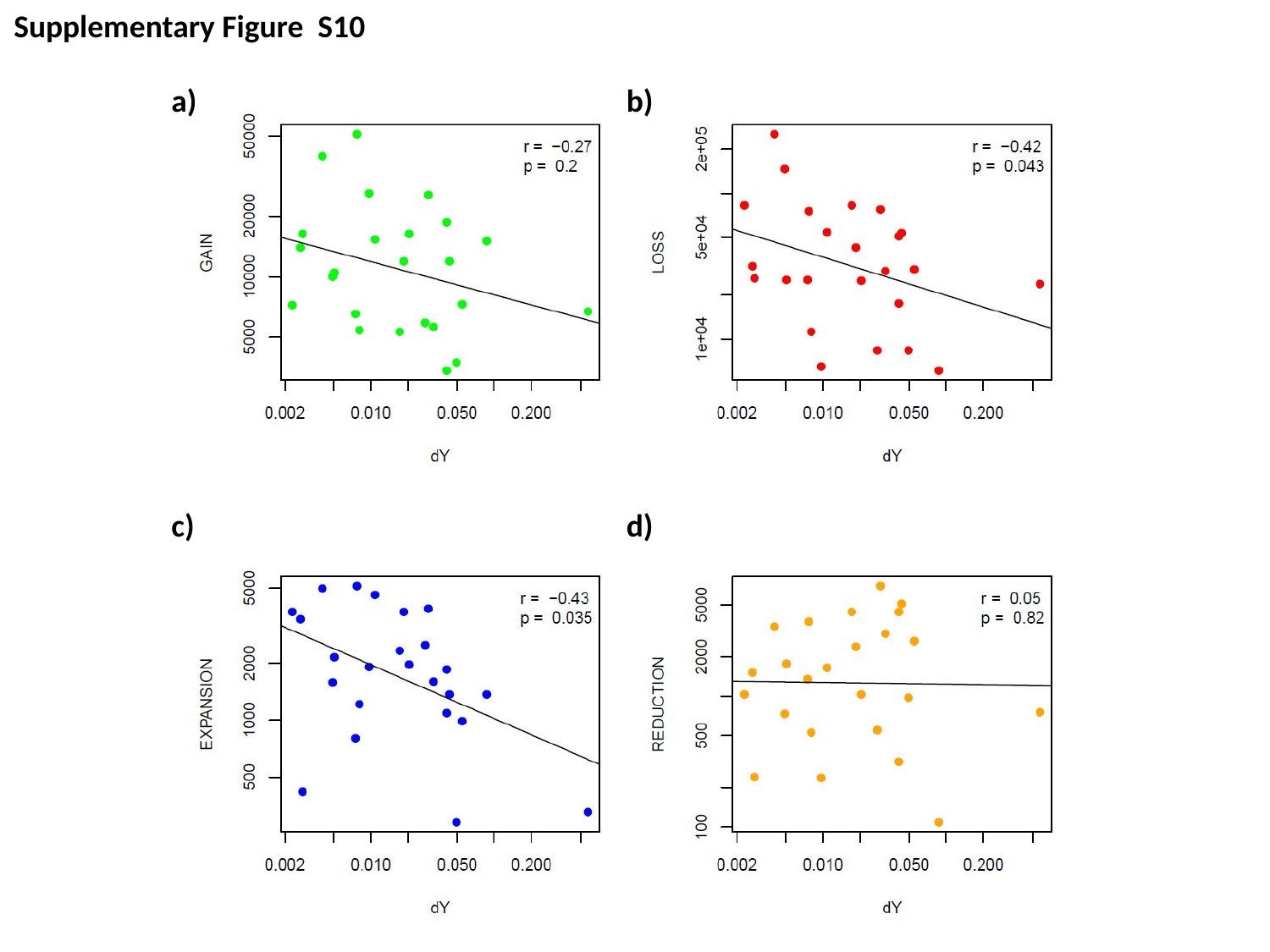

Supplementary Figure S10
a)
b)
c)
d)

## Slide 11
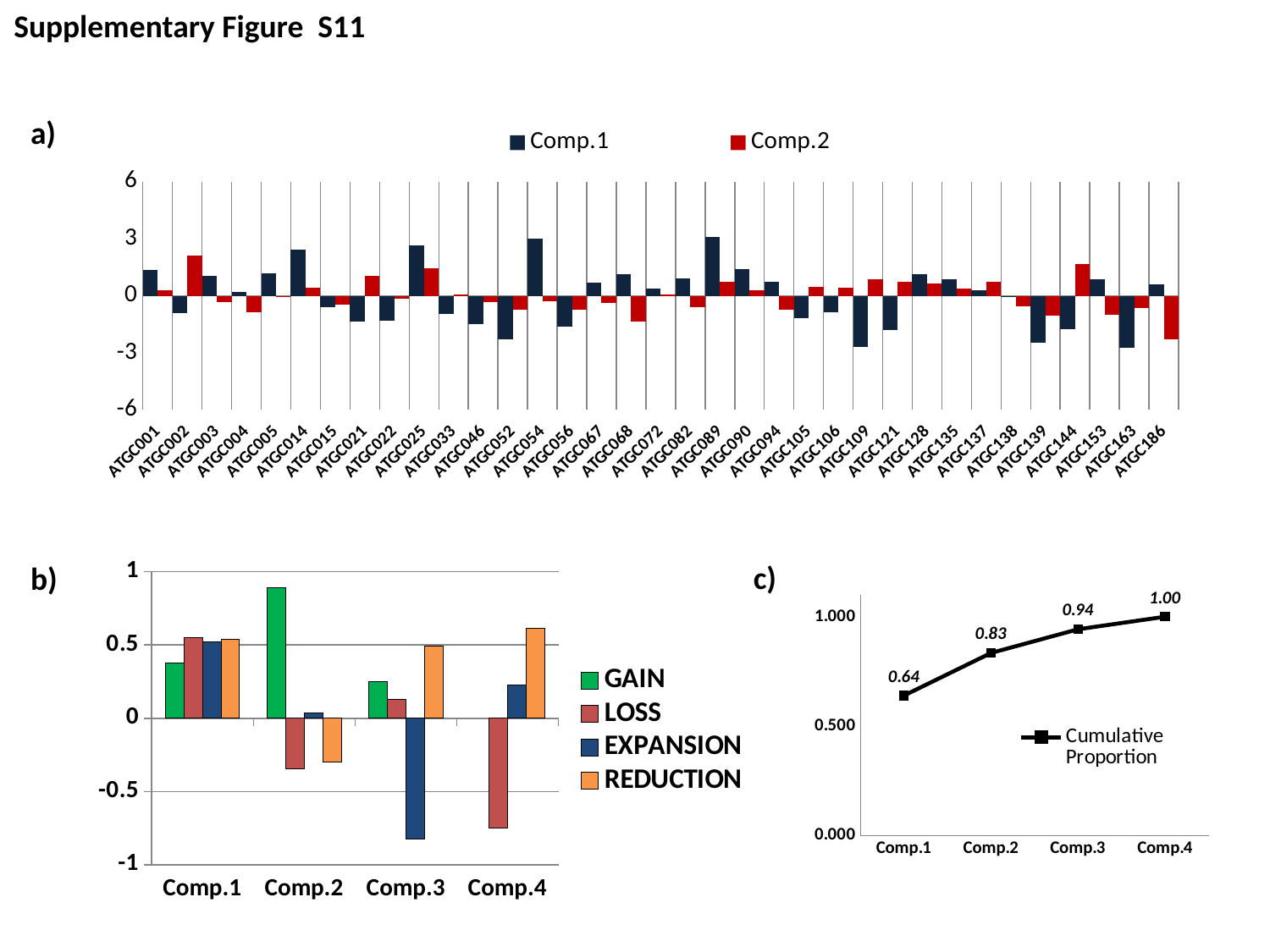

Supplementary Figure S11
a)
### Chart
| Category | Comp.1 | Comp.2 |
|---|---|---|
| ATGC001 | 1.3609452 | 0.274857333 |
| ATGC002 | -0.8984731 | 2.11725291 |
| ATGC003 | 1.0396577 | -0.324935153 |
| ATGC004 | 0.1977347 | -0.864000653 |
| ATGC005 | 1.2058299 | 0.002846699 |
| ATGC014 | 2.422122 | 0.425630267 |
| ATGC015 | -0.610798 | -0.467454201 |
| ATGC021 | -1.3430661 | 1.029061322 |
| ATGC022 | -1.3076038 | -0.148003723 |
| ATGC025 | 2.6346955 | 1.430177461 |
| ATGC033 | -0.9522126 | 0.064164827 |
| ATGC046 | -1.4774159 | -0.309116472 |
| ATGC052 | -2.2836052 | -0.719902454 |
| ATGC054 | 3.0228559 | -0.299704773 |
| ATGC056 | -1.6019819 | -0.722249918 |
| ATGC067 | 0.690804 | -0.360993575 |
| ATGC068 | 1.1458099 | -1.361900275 |
| ATGC072 | 0.3843493 | 0.066997354 |
| ATGC082 | 0.9261443 | -0.604252167 |
| ATGC089 | 3.1048057 | 0.744817933 |
| ATGC090 | 1.4182824 | 0.316263143 |
| ATGC094 | 0.7221407 | -0.72877022 |
| ATGC105 | -1.1732017 | 0.479141026 |
| ATGC106 | -0.8581875 | 0.428190184 |
| ATGC109 | -2.6976898 | 0.860968243 |
| ATGC121 | -1.8021556 | 0.738123852 |
| ATGC128 | 1.1449945 | 0.632088602 |
| ATGC135 | 0.8616172 | 0.401336451 |
| ATGC137 | 0.2913127 | 0.729285644 |
| ATGC138 | -0.0250271 | -0.556919702 |
| ATGC139 | -2.4741186 | -1.054153744 |
| ATGC144 | -1.7723048 | 1.673202202 |
| ATGC153 | 0.8716674 | -0.980518037 |
| ATGC163 | -2.7507247 | -0.645309086 |
| ATGC186 | 0.5827974 | -2.266221302 |c)
### Chart
| Category | Cumulative Proportion |
|---|---|
| Comp.1 | 0.6400751 |
| Comp.2 | 0.8345923 |
| Comp.3 | 0.942704 |
| Comp.4 | 1.0 |
### Chart
| Category | GAIN | LOSS | EXPANSION | REDUCTION |
|---|---|---|---|---|
| Comp.1 | 0.374 | 0.549 | 0.521 | 0.537 |
| Comp.2 | 0.888 | -0.346 | 0.0384 | -0.301 |
| Comp.3 | 0.252 | 0.126 | -0.823 | 0.494 |
| Comp.4 | None | -0.75 | 0.225 | 0.614 |b)

## Slide 12
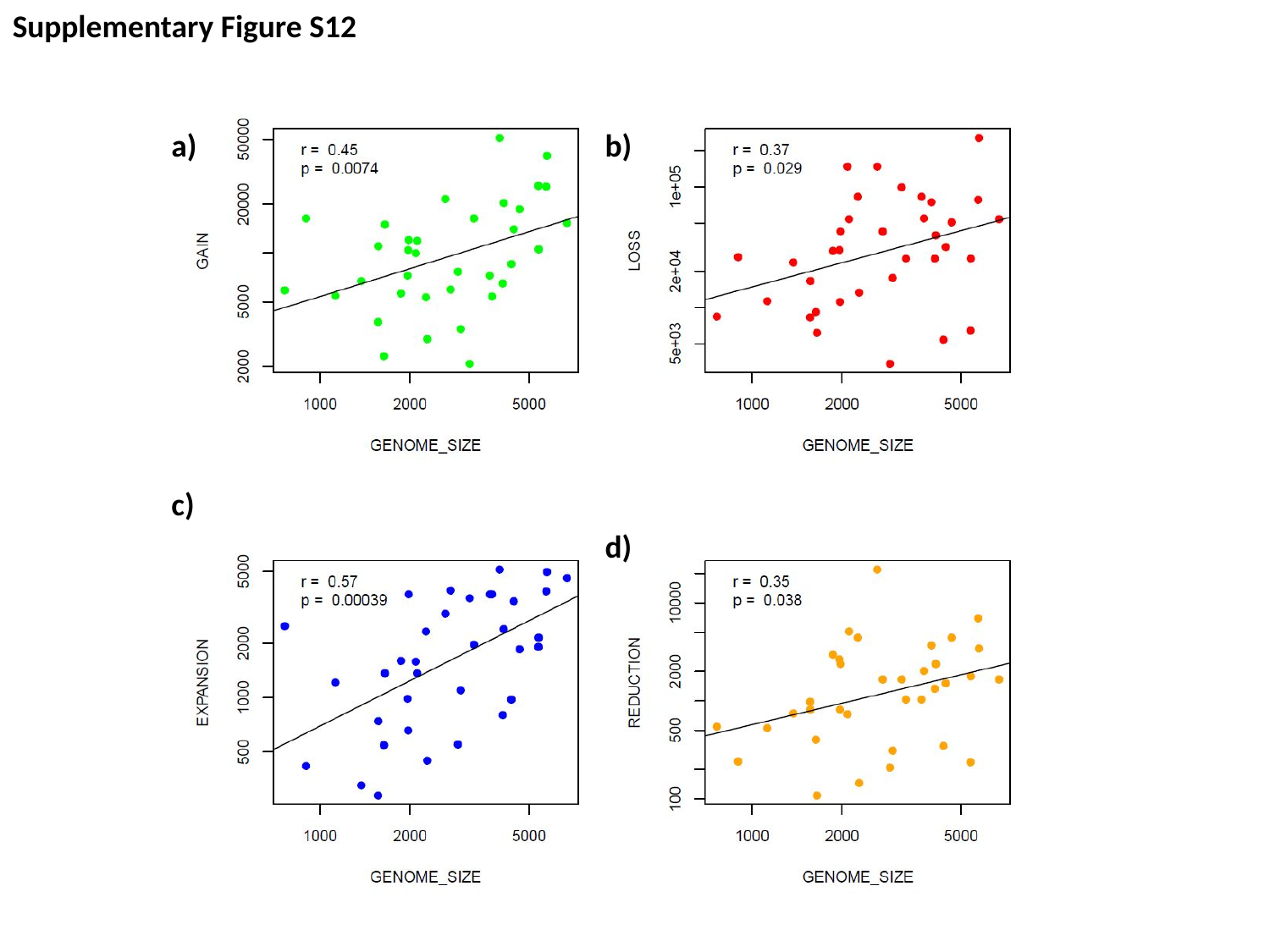

Supplementary Figure S12
a)
b)
c)
d)

## Slide 13
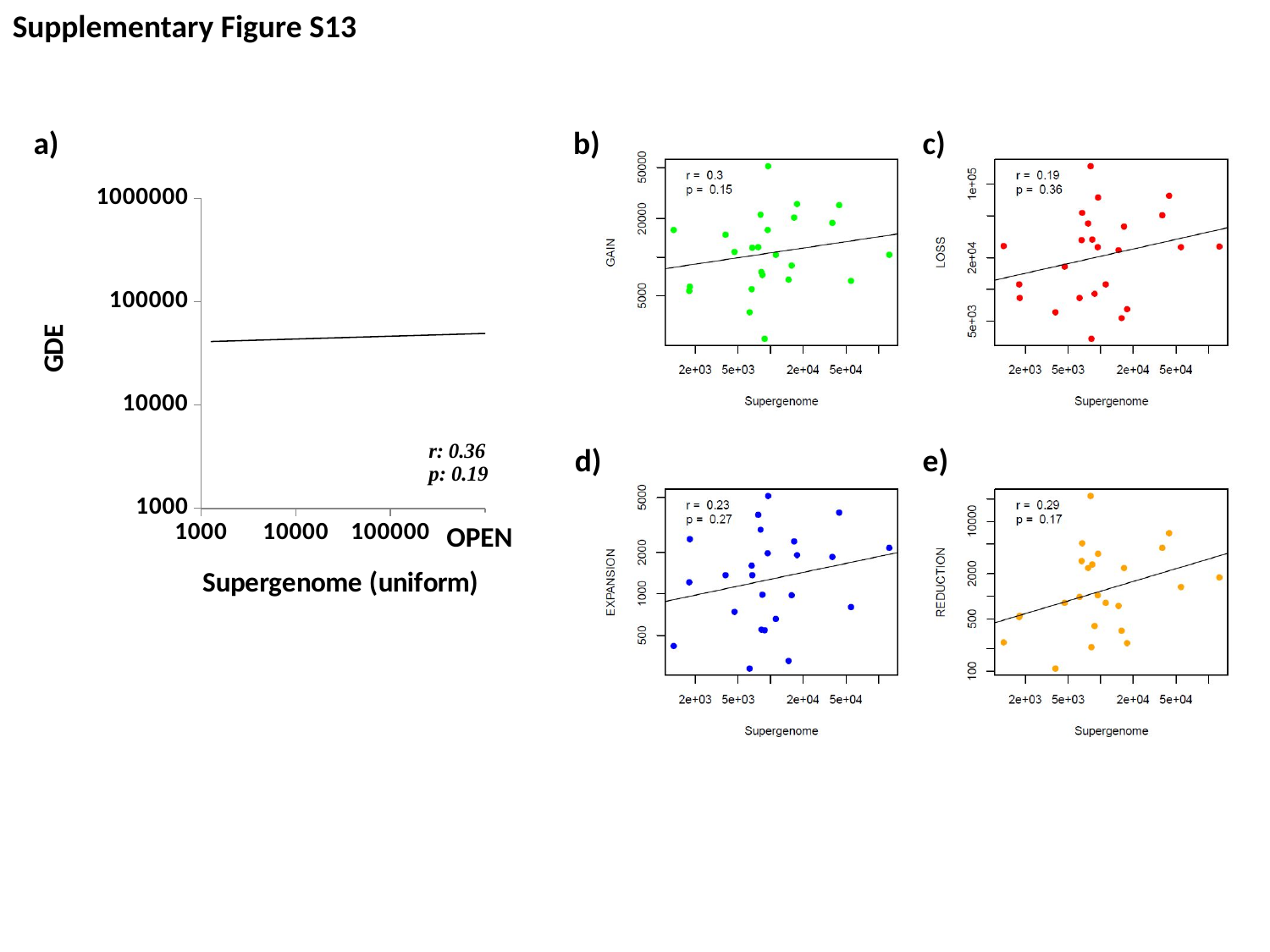

Supplementary Figure S13
a)
b)
c)
### Chart
| Category | Supergenome (uniform) |
|---|---|d)
e)
OPEN

## Slide 14
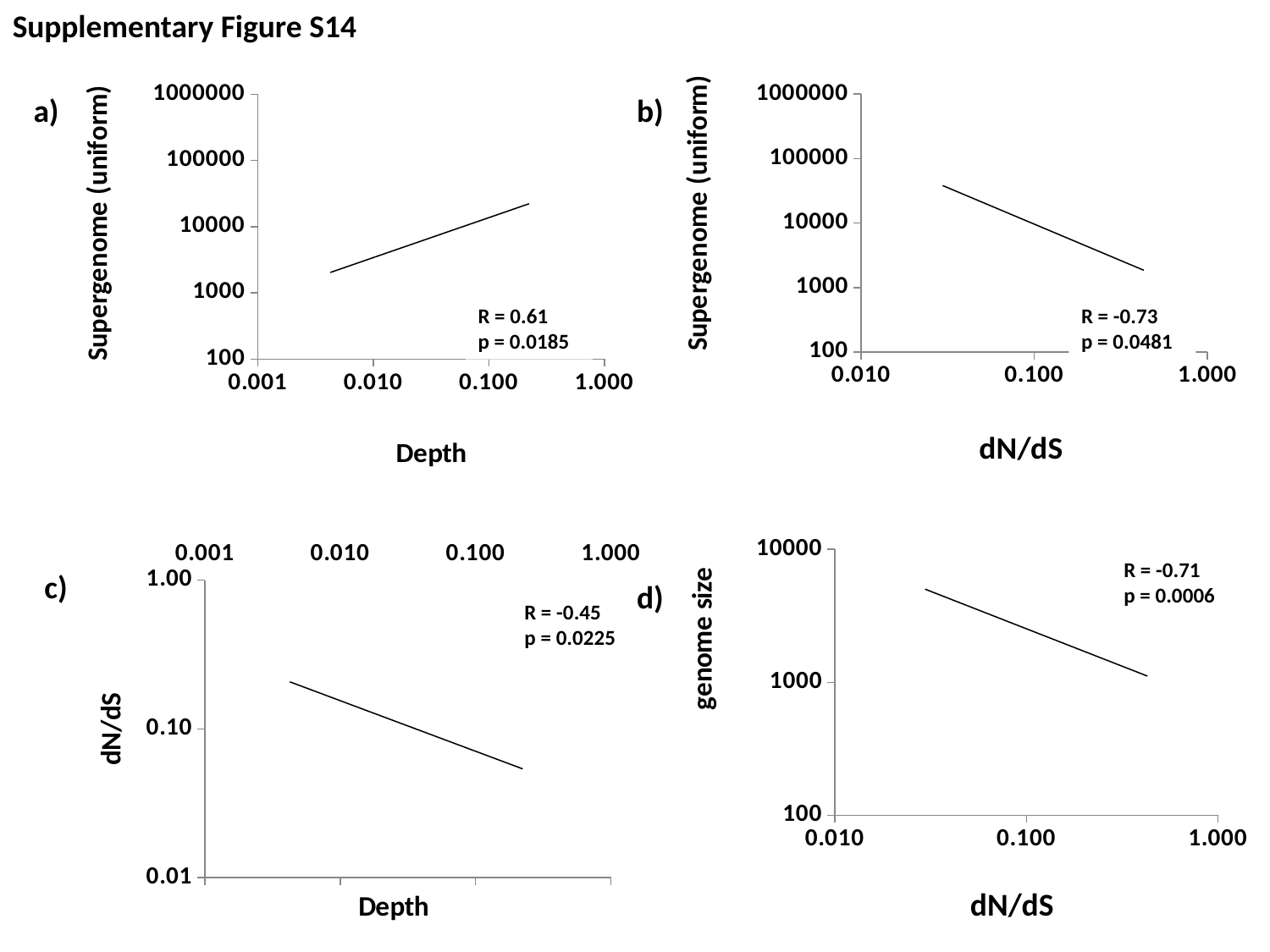

Supplementary Figure S14
### Chart
| Category | |
|---|---|
### Chart
| Category | |
|---|---|a)
b)
R = 0.61
p = 0.0185
R = -0.73
p = 0.0481
### Chart
| Category | |
|---|---|
### Chart
| Category | |
|---|---|R = -0.71
p = 0.0006
c)
d)
R = -0.45
p = 0.0225

## Slide 15
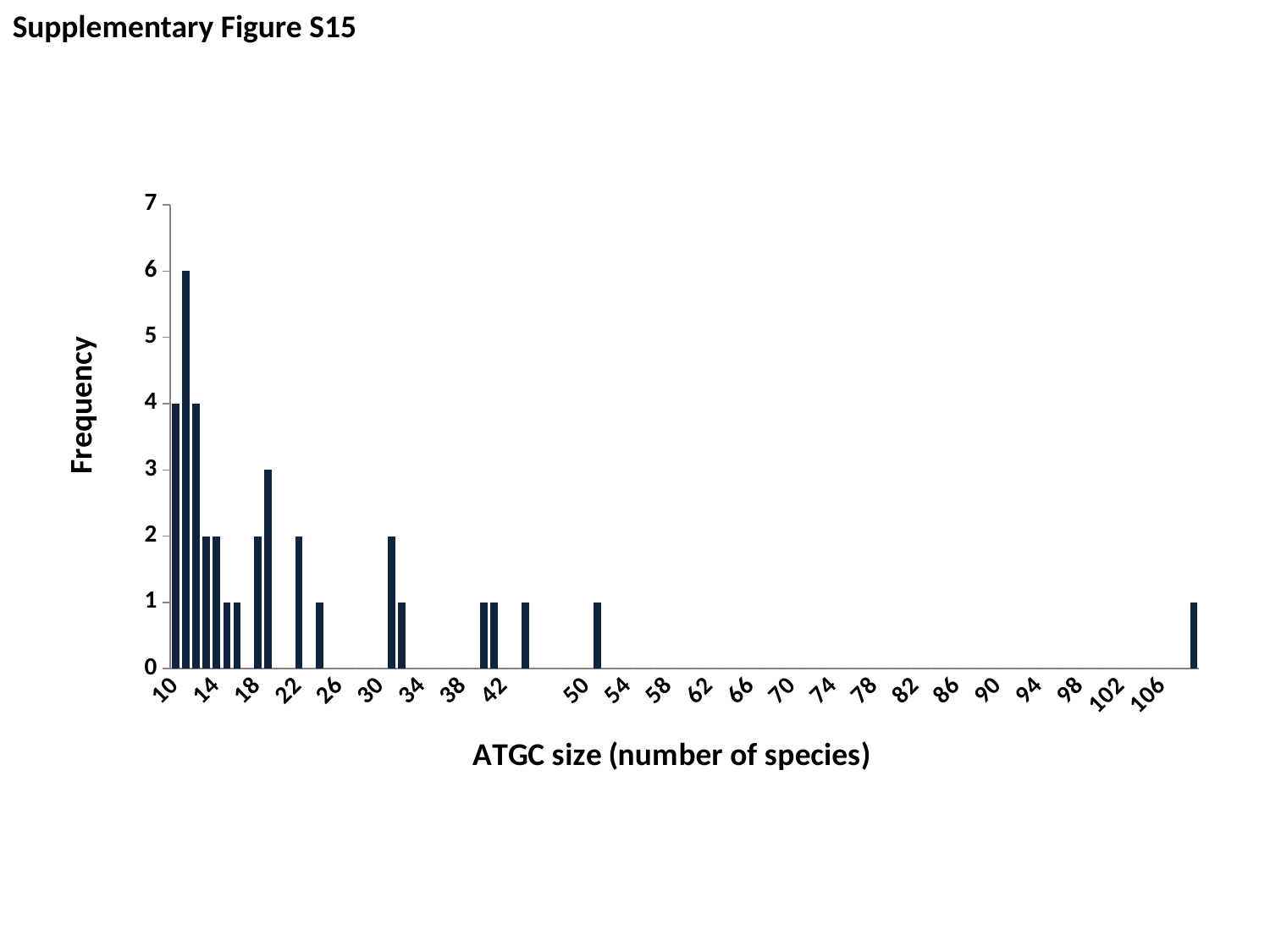

Supplementary Figure S15
### Chart
| Category | |
|---|---|
| 10 | 4.0 |
| 11 | 6.0 |
| 12 | 4.0 |
| 13 | 2.0 |
| 14 | 2.0 |
| 15 | 1.0 |
| 16 | 1.0 |
| 17 | 0.0 |
| 18 | 2.0 |
| 19 | 3.0 |
| 20 | 0.0 |
| 21 | 0.0 |
| 22 | 2.0 |
| 23 | 0.0 |
| 24 | 1.0 |
| 25 | 0.0 |
| 26 | 0.0 |
| 27 | 0.0 |
| 28 | 0.0 |
| 29 | 0.0 |
| 30 | 0.0 |
| 31 | 2.0 |
| 32 | 1.0 |
| 33 | 0.0 |
| 34 | 0.0 |
| 35 | 0.0 |
| 36 | 0.0 |
| 37 | 0.0 |
| 38 | 0.0 |
| 39 | 0.0 |
| 40 | 1.0 |
| 41 | 1.0 |
| 42 | 0.0 |
| 43 | 0.0 |
| 44 | 1.0 |
| | None |
| | None |
| | None |
| 48 | 0.0 |
| 49 | 0.0 |
| 50 | 0.0 |
| 51 | 1.0 |
| 52 | 0.0 |
| 53 | 0.0 |
| 54 | 0.0 |
| 55 | 0.0 |
| 56 | 0.0 |
| 57 | 0.0 |
| 58 | 0.0 |
| 59 | 0.0 |
| 60 | 0.0 |
| 61 | 0.0 |
| 62 | 0.0 |
| 63 | 0.0 |
| 64 | 0.0 |
| 65 | 0.0 |
| 66 | 0.0 |
| 67 | 0.0 |
| 68 | 0.0 |
| 69 | 0.0 |
| 70 | 0.0 |
| 71 | 0.0 |
| 72 | 0.0 |
| 73 | 0.0 |
| 74 | 0.0 |
| 75 | 0.0 |
| 76 | 0.0 |
| 77 | 0.0 |
| 78 | 0.0 |
| 79 | 0.0 |
| 80 | 0.0 |
| 81 | 0.0 |
| 82 | 0.0 |
| 83 | 0.0 |
| 84 | 0.0 |
| 85 | 0.0 |
| 86 | 0.0 |
| 87 | 0.0 |
| 88 | 0.0 |
| 89 | 0.0 |
| 90 | 0.0 |
| 91 | 0.0 |
| 92 | 0.0 |
| 93 | 0.0 |
| 94 | 0.0 |
| 95 | 0.0 |
| 96 | 0.0 |
| 97 | 0.0 |
| 98 | 0.0 |
| 99 | 0.0 |
| 100 | 0.0 |
| 101 | 0.0 |
| 102 | 0.0 |
| 103 | 0.0 |
| 104 | 0.0 |
| 105 | 0.0 |
| 106 | 0.0 |
| 107 | 0.0 |
| 108 | 0.0 |
| 109 | 1.0 |

## Slide 16
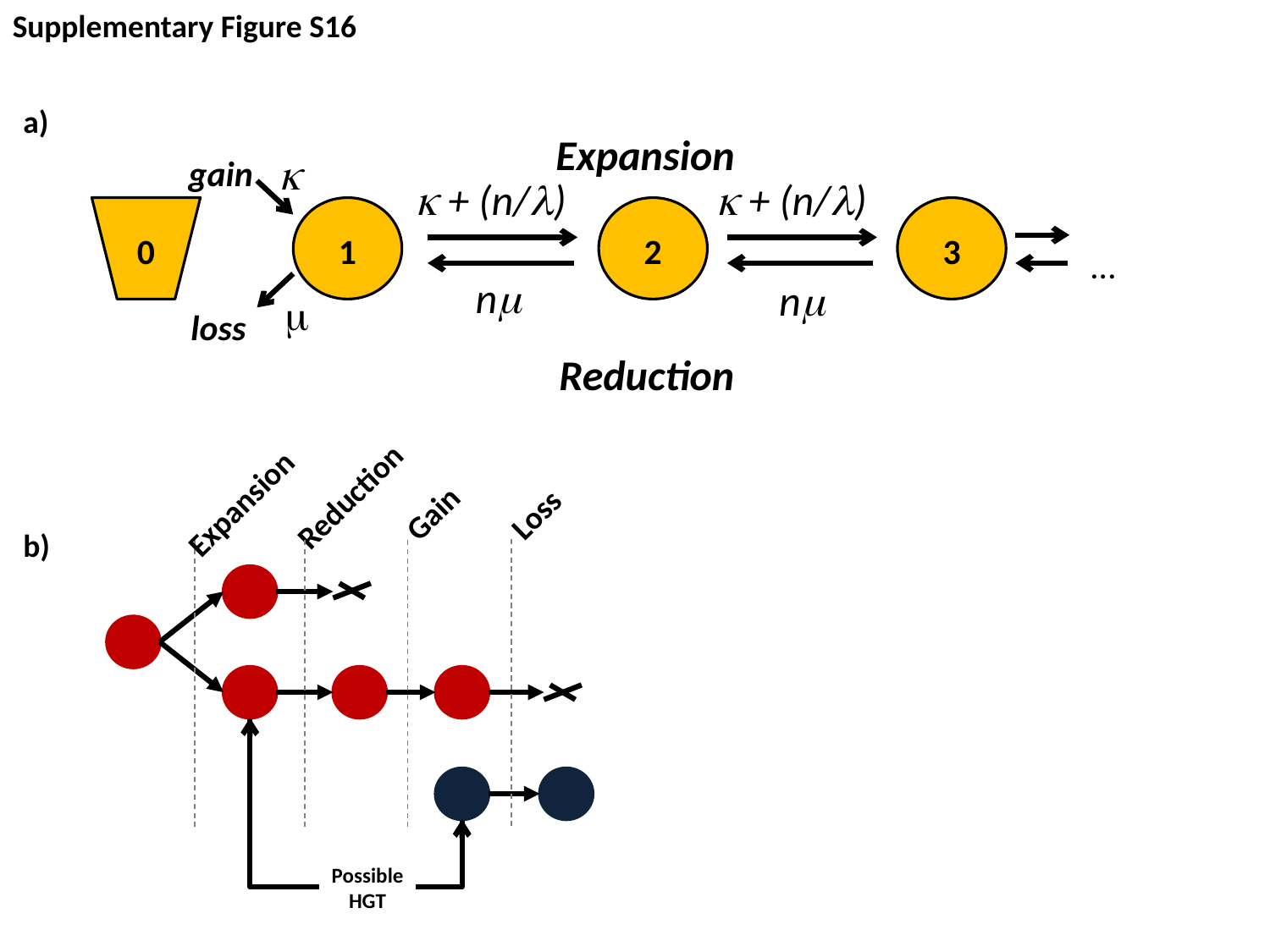

Supplementary Figure S16
a)
Expansion

gain
 + (n/λ)
 + (n/λ)
0
1
2
3
…
n
n

loss
Reduction
Reduction
Expansion
Gain
Loss
Possible
HGT
b)
