# Supplementary material for: Genomes in turmoil: quantification of genome dynamics in prokaryote supergenomes
Source: BMC Biol. 2014 Aug 21;12:66. doi: 10.1186/s12915-014-0066-4 (PMC4166000; doi:10.1186/s12915-014-0066-4)
Supplement: Additional file 3: Table S2. — Characteristics of ATGCs. [file 12915_2014_66_MOESM3_ESM.pdf]

ATGC	GENERA	Total Branch Length	Tree Depth	Total inferred number				Rate per substitution per site				Rate per substitution per gene				Principal components				Total Flux rate	Balance Ratio
				Gain	Loss	Expansion	Reduction	Gain	Loss	Expansion	Reduction	Gain	Loss	Expansion	Reduction	PC1	PC2	PC3	PC4		
ATGC001	Citrobacter-Enterobacter-Escherichia-Salmonella-Shigella	1.689	0.183	31359	85342	3149	7441	18562.6	50516.8	1863.8	4404.7	17.306	47.098	1.738	4.107	-1.361	0.275	0.694	0.238	75348	0.372
ATGC002	Enterobacter-Klebsiella	0.350	0.066	9050	2256	670	83	25865.7	6447.7	1914.3	236.7	24.475	6.101	1.811	0.224	0.898	2.117	-0.711	0.165	34464	4.156
ATGC003	Streptococcus	0.208	0.067	2479	11153	285	1061	11893.5	53500.2	1368.4	5091.6	11.783	53.004	1.356	5.044	-1.040	-0.325	0.922	0.244	71854	0.226
ATGC004	Streptococcus	0.264	0.094	1485	7707	422	787	5632.8	29232.9	1600.8	2984.4	5.500	28.543	1.563	2.914	-0.198	-0.864	0.212	0.540	39451	0.225
ATGC005	Streptococcus	0.106	0.023	1260	4456	396	251	11939.7	42215.0	3755.0	2379.5	11.322	40.030	3.561	2.256	-1.206	0.003	-0.439	0.292	60289	0.352
ATGC014	Bacillus	0.459	0.073	11687	35535	1786	3168	25485.4	77488.3	3895.5	6908.0	25.539	77.651	3.904	6.922	-2.422	0.426	0.305	0.325	113777	0.348
ATGC015	Bacillus	0.658	0.223	4292	16649	529	884	6521.1	25293.2	803.6	1342.8	6.562	25.453	0.809	1.351	0.611	-0.467	0.596	0.018	33961	0.275
ATGC021	Chlamydia	0.029	0.004	471	748	12	7	16346.1	25967.5	418.8	240.0	14.033	22.293	0.360	0.206	1.343	1.029	0.833	-1.202	42972	0.640
ATGC022	Chlamydia-Chlamydophila	0.116	0.046	628	1295	140	61	5434.3	11201.6	1212.8	524.3	5.232	10.784	1.168	0.505	1.308	-0.148	-0.376	0.252	18373	0.567
ATGC025	Mycobacterium	0.110	0.007	5579	8218	561	407	50822.9	74867.5	5109.4	3708.1	45.888	67.598	4.613	3.348	-2.635	1.430	-0.001	0.010	134508	0.712
ATGC033	Mycoplasma	0.026	0.013	152	216	64	14	5864.8	8335.1	2483.7	547.6	4.611	6.553	1.953	0.431	0.952	0.064	-1.086	0.678	17231	0.940
ATGC046	Rickettsia	0.482	0.097	3222	11422	157	362	6677.0	23673.5	324.6	750.0	7.440	26.379	0.362	0.836	1.477	-0.309	1.258	-0.492	31425	0.287
ATGC052	Helicobacter	1.157	0.117	4320	9597	333	1127	3732.7	8293.4	287.7	973.6	3.584	7.964	0.276	0.935	2.284	-0.720	1.164	0.450	13287	0.434
ATGC054	Staphylococcus	0.085	0.014	1834	12525	249	1849	21511.8	146937.4	2923.8	21695.6	19.886	135.834	2.703	20.056	-3.023	-0.300	1.098	0.404	193069	0.145
ATGC056	Lactobacillus	0.316	0.147	1078	5540	345	99	3417.1	17553.4	1093.8	313.6	3.341	17.160	1.069	0.307	1.602	-0.722	-0.591	-0.316	22378	0.252
ATGC067	Corynebacterium	0.025	0.009	253	3690	40	19	10013.0	146236.3	1581.7	734.4	9.006	131.524	1.423	0.661	-0.691	-0.361	0.022	-1.447	158565	0.079
ATGC068	Corynebacterium	0.081	0.012	431	6691	188	356	5337.6	82891.0	2334.8	4413.8	4.894	76.005	2.141	4.047	-1.146	-1.362	0.109	0.097	94977	0.088
ATGC072	Pseudomonas	0.550	0.107	5760	14042	1190	972	10466.9	25516.1	2162.0	1766.2	9.248	22.545	1.910	1.561	-0.384	0.067	-0.119	0.368	39911	0.463
ATGC082	Clostridium	0.117	0.024	843	9655	437	120	7222.7	82676.3	3745.0	1030.4	6.623	75.815	3.434	0.945	-0.926	-0.604	-0.878	-0.576	94674	0.131
ATGC089	Burkholderia	0.117	0.024	4630	29516	583	400	39592.8	252405.6	4988.0	3417.1	35.492	226.265	4.471	3.063	-3.105	0.745	0.053	-0.894	300403	0.174
ATGC090	Burkholderia	0.389	0.070	5936	21057	1781	639	15276.1	54191.8	4582.4	1643.7	13.586	48.196	4.075	1.462	-1.418	0.316	-0.682	-0.061	75694	0.356
ATGC094	Sulfolobus	0.162	0.071	963	6893	635	266	5955.8	42639.2	3930.5	1643.1	6.064	43.415	4.002	1.673	-0.722	-0.729	-0.876	0.190	54169	0.223
ATGC105	Bifidobacterium	0.287	0.117	2983	3202	188	235	10383.5	11147.5	654.8	817.4	8.342	8.955	0.526	0.657	1.173	0.479	0.648	0.239	23003	0.923
ATGC106	Bifidobacterium	0.047	0.023	523	781	35	38	11039.4	16473.2	742.2	811.7	8.922	13.314	0.600	0.656	0.858	0.428	0.588	-0.022	29067	0.682
ATGC109	Listeria	0.656	0.168	5021	2239	362	136	7650.2	3411.0	551.3	207.2	7.536	3.360	0.543	0.204	2.698	0.861	-0.009	0.371	11820	2.267
ATGC121	Shewanella	0.644	0.172	5522	3438	626	224	8575.7	5339.8	972.9	348.5	7.295	4.543	0.828	0.296	1.802	0.738	-0.265	0.459	15237	1.679
ATGC128	Yersinia	0.238	0.102	4821	9403	570	564	20234.4	39460.0	2390.6	2368.8	17.546	34.216	2.073	2.054	-1.145	0.632	0.181	0.149	64454	0.541
ATGC135	Xanthomonas	0.324	0.124	4534	10206	1106	492	13992.6	31495.5	3412.7	1518.1	12.153	27.354	2.964	1.319	-0.862	0.401	-0.516	0.223	50419	0.527
ATGC137	Brucella-Ochrobactrum	0.233	0.113	3798	5877	459	241	16268.2	25176.9	1968.2	1033.0	15.603	24.148	1.888	0.991	-0.291	0.729	-0.104	0.014	44446	0.696
ATGC138	Neisseria	0.260	0.033	1889	7741	257	685	7277.9	29817.4	990.4	2637.8	6.432	26.352	0.875	2.331	0.025	-0.557	0.730	0.297	40723	0.255
ATGC139	Francisella	0.511	0.147	1187	4637	279	205	2324.1	9075.1	545.7	401.2	1.981	7.737	0.465	0.342	2.474	-1.054	-0.001	0.153	12346	0.303
ATGC144	Campylobacter	0.108	0.037	1613	655	147	12	14996.7	6085.1	1364.5	109.2	14.606	5.926	1.329	0.106	1.772	1.673	-0.890	-0.224	22556	2.641
ATGC153	Acinetobacter	0.258	0.064	1397	14040	966	523	5416.2	54453.7	3746.6	2029.0	5.142	51.694	3.557	1.926	-0.872	-0.981	-0.740	0.122	65645	0.162
ATGC163	Propionibacterium	0.268	0.132	789	3540	119	39	2947.5	13222.7	446.0	145.0	2.668	11.970	0.404	0.131	2.751	-0.645	-0.104	-0.742	16761	0.254
ATGC186	Legionella	0.088	0.017	183	8704	311	144	2082.5	99232.1	3545.5	1646.9	1.886	89.856	3.211	1.491	-0.583	-2.266	-1.022	-0.321	106507	0.056

ATGC	Pangenome	Number of non-ancestral gains	Number of multiple non-ancestral gains	Supergenome estimate, uniform distribution			Supergenome estimate, power law distribution			Supergenome estimate, binomial mixture		Supergenome estimate, capture-recapture	Number of genomes	Median genome Size			Median GC content	Median dN/dS	Synteny distance (dY) at 0.01 dNT	Lifestyle
				Size	Confidence interval width	Relative to genome size	Size	Exponent	Relative to genome size	Size	Number of cate-gories			bp	Protein-coding genes	Gene Families				
ATGC001	24845	28950	10059	37267	458	8.84	45092	0.44	10.70	33452	6	55175	109	4989733	4652	4215	0.528	0.045	0.04255	facultative host-associated
ATGC002	10802	8922	2429	17560	429	3.35	18403	0.23	3.52	14326	4	17357	11	5680456	5375	5234	0.583	0.039	0.00819	facultative host-associated
ATGC003	4478	2117	482	6754	297	3.34	6754	0.00	3.34	5907	6	7051	22	2133789	2114	2020	0.408	0.182	0.04313	free-living
ATGC004	3746	1209	167	6664	554	3.69	6664	0.00	3.69	5872	5	5332	22	1910066	1865	1808	0.395	0.176	0.03210	free-living
ATGC005	3466	1186	124	7669	877	3.94	7669	0.00	3.94	4688	5	5814	16	2084900	1977	1948	0.422	0.093	0.01850	free-living
ATGC014	16678	10985	1514	43302	1676	7.83	43303	0.00	7.83	26600	6	37108	31	5691541	5704	5533	0.361	0.054	0.02911	free-living
ATGC015	9242	4094	162	55655	7699	14.00	78848	0.36	19.83	67131	7	18605	24	4063283	4089	3976	0.459	0.058	0.01422	free-living
ATGC021	1168	439	193	1260	31	1.40	1344	0.51	1.49	1303	5	1567	44	1046020	898	901	0.417	0.429	0.00275	intracellular parasite
ATGC022	1441	536	163	1758	83	1.74	1759	0.00	1.74	1576	5	1792	19	1169029	1126	1011	0.397	0.400	0.00840	intracellular parasite
ATGC025	7293	5264	1850	9484	190	2.47	13514	0.63	3.52	13106	6	17474	32	4409130	3981	3836	0.659		0.03113	facultative host-associated
ATGC033	883	178	14	1784	539	2.40	1785	0.00	2.41	901	3	901	11	968554	762	742	0.319			facultative host-associated
ATGC046	4454	3109	344	14677	1293	13.26	24011	0.47	21.69	7280	6	11509	40	1233088	1374	1107	0.322	0.117	0.05306	intracellular parasite
ATGC052	4568	3978	1351	6377	179	4.25	7246	0.40	4.83	8632	6	11675	51	1628207	1564	1501	0.397	0.160	0.00712	facultative host-associated
ATGC054	4815	1917	334	8071	464	3.29	8322	0.20	3.39	5792	7	7074	41	2837953	2624	2452	0.337	0.100	0.04813	free-living
ATGC056	4893	1055	0	open						5784	4	6340	10	3024728	2957	2896	0.473	0.033	0.04546	free-living
ATGC067	2721	287	0	open						3144	5	3588	15	2322675	2089	2074	0.530	0.136	0.00693	free-living
ATGC068	3453	499	0	open						3889	4	4519	12	2466919	2262	2227	0.543	0.111	0.01556	free-living
ATGC072	11389	5603	129	125699	20767	23.73	125700	0.00	23.73	24983	5	28686	12	6095696	5386	5297	0.633	0.033	0.02630	free-living
ATGC082	5993	1007	0	open						9718	5	8607	10	4025029	3691	3655	0.292	0.099	0.01886	free-living
ATGC089	13381	4901	12	open						30825	5	30581	13	6406482	5743	5707	0.688	0.077	0.05573	free-living
ATGC090	14540	6508	0	open						23983	5	28567	12	7530187	6697	6428	0.678	0.057	0.00406	free-living
ATGC094	4471	986	0	open						5629	5	6483	12	2686155	2735	2638	0.356	0.231	0.07020	free-living
ATGC105	4352	2913	411	11174	829	5.72	11174	0.00	5.72	30285	5	6607	11	2454110	1972	1952	0.609	0.086	0.07281	facultative host-associated
ATGC106	2018	452	32	4631	1020	2.95	5619	0.37	3.58	2221	4	2363	10	1938894	1567	1568	0.615			facultative host-associated
ATGC109	5834	4899	1623	8207	210	2.89	11712	0.59	4.12	6625	6	6881	31	2934366	2891	2843	0.385	0.030	0.01692	free-living
ATGC121	8090	5274	1024	15597	633	3.74	15929	0.16	3.82	58299	5	13057	14	5125160	4360	4166	0.475	0.045	0.02065	free-living
ATGC128	8287	4421	729	16479	778	4.19	20747	0.41	5.27	100790	5	14270	19	4744469	4114	3934	0.489		0.04346	facultative host-associated
ATGC135	9814	5005	0	open						65198	4	21460	13	5119661	4447	4204	0.654	0.060	0.04880	facultative host-associated
ATGC137	6012	3454	829	9376	343	2.92	11086	0.41	3.45	20891	5	20621	19	3407297	3268	3212	0.584		0.04154	facultative host-associated
ATGC138	4344	1614	217	8395	670	4.33	8395	0.00	4.33	7985	6	7640	18	2218847	1961	1937	0.537	0.088	0.02045	facultative host-associated
ATGC139	3247	1071	83	8801	1360	5.46	8801	0.00	5.46	3845	5	4805	18	1916517	1634	1613	0.333	0.111	0.06210	facultative host-associated
ATGC144	2637	1548	421	3811	179	2.31	3811	0.00	2.31	2969	4	3537	11	1690063	1646	1650	0.309	0.070	0.00597	facultative host-associated
ATGC153	6455	1320	0	open			open			34720	7	10539	14	3959662	3759	3517	0.403	0.038	0.05374	free-living
ATGC163	3485	602	0	open			open			5911	5	6198	11	2518661	2280	2270	0.608		0.01601	free-living
ATGC186	4587	1	0	open			open			5585	4	6424	10	3496368	3166	3038	0.391	0.095	0.03922	facultative host-associated
