# Supplementary material for: Genomes in turmoil: quantification of genome dynamics in prokaryote supergenomes
Source: BMC Biol. 2014 Aug 21;12:66. doi: 10.1186/s12915-014-0066-4 (PMC4166000; doi:10.1186/s12915-014-0066-4)
Supplement: Additional file 4: Table S3. — Comparison of the present supergenome size estimates with previously published estimates. [file 12915_2014_66_MOESM4_ESM.docx]

**Table S3.** Comparison of the present supergenome size estimates with previously published estimates

| **GENERA** | **ATGC** | **S, uniform** | **S, power** | **Previous estimates** | **Reference** |
| --- | --- | --- | --- | --- | --- |
| *Enterobacteria* | ATGC001 | 9.7 | 13.2 | 5.6-9.4-open | {Snipen, 2009 #4747}  {Tettelin, 2008 #4739}  {Gordienko, 2013 #4755} |
| *Enterobacter-Klebsiella* | ATGC002 | 5.6 | 7.8 |  |  |
| *Streptococcus* | ATGC003 | 7.3 | 8.1 | 2.6-open | {Snipen, 2009 #4747}  {Tettelin, 2008 #4739} |
| *Streptococcus* | ATGC004 | 11.4 | 13.4 | 3.4 | {Snipen, 2009 #4747} |
| *Streptococcus* | ATGC005 | 14.8 | 17.7 |  |  |
| *Bacillus* | ATGC014 | 10.8 | 12.4 | 6.3 - open | {Snipen, 2009 #4747}  {Tettelin, 2008 #4739} |
| *Bacillus* | ATGC015 | 18.6 | 37.2 |  |  |
| *Chlamydia* | ATGC021 | 4.2 | 43.0 |  |  |
| *Chlamydia-Chlamydophila* | ATGC022 | 4.9 | 6.2 |  |  |
| *Mycobacterium* | ATGC025 | 4.5 | 18.1 |  |  |
| *Mycoplasma* | ATGC033 | 32.7 | open |  |  |
| *Rickettsia* | ATGC046 | 17.8 | 32.5 |  |  |
| *Helicobacter* | ATGC052 | 6.2 | 10.8 | 2.7 | {Snipen, 2009 #4747} |
| *Staphylococcus* | ATGC054 | 9.2 | 12.6 | 3.3 | {Snipen, 2009 #4747} |
| *Lactobacillus* | ATGC056 | open | open | 3.2 | {Broadbent, 2012 #4756} |
| *Corynebacterium* | ATGC067 | open | open | open | {Soares, 2013 #4753} |
| *Corynebacterium* | ATGC068 | open | open |  |  |
| *Pseudomonas* | ATGC072 | 20.3 | open |  |  |
| *Clostridium* | ATGC082 | open | open | 4.5 | {Snipen, 2009 #4747} |
| *Burkholderia* | ATGC089 | open | open | 3.1 | {Ussery, 2009 #4759} |
| *Burkholderia* | ATGC090 | open | open | 3.3 | {Ussery, 2009 #4759} |
| *Sulfolobus* | ATGC094 | open | open |  |  |
| *Bifidobacterium* | ATGC105 | 9.2 | 9.9 | 1.9- open | {Bottacini, 2010 #4750}  {Bottacini, 2014 #4751} |
| *Bifidobacterium* | ATGC106 | 19.6 | 88.4 |  |  |
| *Listeria* | ATGC109 | 4.8 | 14.0 | open | {Kuenne, 2013 #4757} |
| *Shewanella* | ATGC121 | 7.2 | 9.2 |  |  |
| *Yersinia* | ATGC128 | 9.2 | 18.9 | 2.3 | {Snipen, 2009 #4747} |
| *Xanthomonas* | ATGC135 | open | open |  |  |
| *Brucella-Ochrobactrum* | ATGC137 | 6.0 | 12.9 |  |  |
| *Neisseria* | ATGC138 | 11.3 | 12.6 | 1.6 | {Budroni, 2011 #4758} |
| *Francisella* | ATGC139 | 19.6 | open | 2.3 | {Snipen, 2009 #4747} |
| *Campylobacter* | ATGC144 | 5.6 | 6.2 | 2.2-3.9 | {Snipen, 2009 #4747}  {Meric, 2014 #4752} |
| *Acinetobacter* | ATGC153 | open | open | 4.4 | {Snipen, 2009 #4747} |
| *Propionibacterium* | ATGC163 | open | open |  |  |
| *Legionella* | ATGC186 | open | open |  |  |
