# Supplementary material for: Genomes in turmoil: quantification of genome dynamics in prokaryote supergenomes
Source: BMC Biol. 2014 Aug 21;12:66. doi: 10.1186/s12915-014-0066-4 (PMC4166000; doi:10.1186/s12915-014-0066-4)
Supplement: Additional file 5: Table S4. — Parameters used in the Count analysis. [file 12915_2014_66_MOESM5_ESM.docx]

**Supplementary Table S4.** Parameters used in the analysis with COUNT

| **COUNT parameters** | **-opt_rounds** | **-max_paralogs** | **-gain_k** | **-loss_k** | **-duplication_k** | **-length_k** |
| --- | --- | --- | --- | --- | --- | --- |
| **ATGC001, ATGC002, ATGC025, ATGC033, ATGC052, ATGC106, ATGC121** | 100 | 1000 | 1 | 2 | 1 | 1 |
| **Other ATGCs** | 100 | 1000 | 2 | 2 | 2 | 1 |
